# Molecular-dynamics-simulation-guided membrane engineering allows the increase of membrane fatty acid chain length in *Saccharomyces cerevisiae*

**DOI:** 10.1038/s41598-021-96757-y

**Published:** 2021-08-30

**Authors:** Jeroen M. Maertens, Simone Scrima, Matteo Lambrughi, Samuel Genheden, Cecilia Trivellin, Leif A. Eriksson, Elena Papaleo, Lisbeth Olsson, Maurizio Bettiga

**Affiliations:** 1 Department of Biology and Biological Engineering, Division of Industrial Biotechnology, Chalmers University of Technology, Kemivägen 10, 412 96 Gothenburg, Sweden; 2 Computational Biology Laboratory, Danish Cancer Society Research Center, Strandboulevarden 49, 2100 Copenhagen, Denmark; 3 Department for Chemistry and Molecular Biology, University of Gothenburg, Medicinaregatan 9c, 40530 Gothenburg, Sweden

**Keywords:** Industrial microbiology, Metabolic engineering, Computational models

## Abstract

The use of lignocellulosic-based fermentation media will be a necessary part of the transition to a circular bio-economy. These media contain many inhibitors to microbial growth, including acetic acid. Under industrially relevant conditions, acetic acid enters the cell predominantly through passive diffusion across the plasma membrane. The lipid composition of the membrane determines the rate of uptake of acetic acid, and thicker, more rigid membranes impede passive diffusion. We hypothesized that the elongation of glycerophospholipid fatty acids would lead to thicker and more rigid membranes, reducing the influx of acetic acid. Molecular dynamics simulations were used to predict the changes in membrane properties. Heterologous expression of *Arabidopsis thaliana* genes fatty acid elongase 1 (*FAE1*) and glycerol-3-phosphate acyltransferase 5 (*GPAT5*) increased the average fatty acid chain length. However, this did not lead to a reduction in the net uptake rate of acetic acid. Despite successful strain engineering, the net uptake rate of acetic acid did not decrease. We suggest that changes in the relative abundance of certain membrane lipid headgroups could mitigate the effect of longer fatty acid chains, resulting in a higher net uptake rate of acetic acid.

## Introduction

The current linear model for resource utilisation and industrial production is unsustainable and must be shifted towards a more circular model^1^. This means that bio-based processes will become increasingly important^2^. Lignocellulosic biomass must be pre-treated and hydrolysed prior to being fermented to produce fuels, platform chemicals and other materials^3^. Although pre-treatment and hydrolysis are essential to release sugars from the biomass, they also result in fermentation medium containing many microbial growth inhibitors^4^.

**Figure 1:**
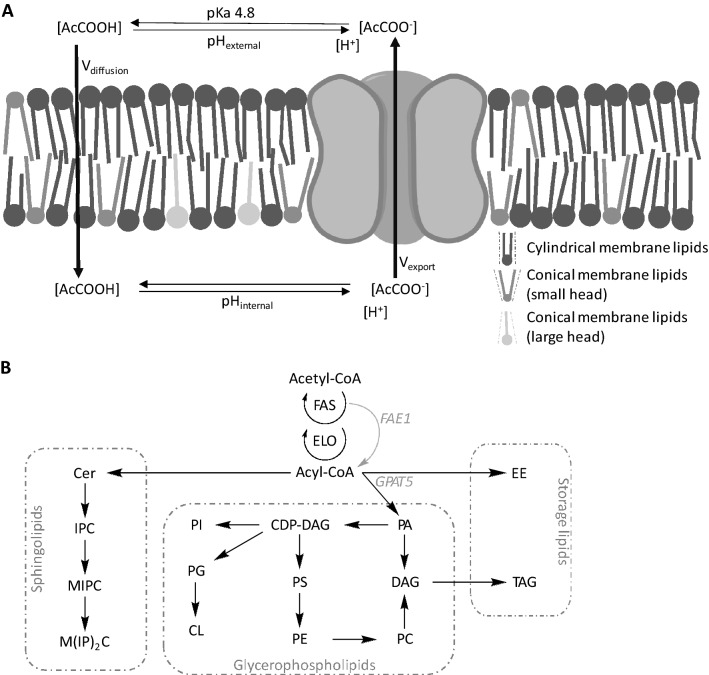
Membrane composition, acetic acid homeostasis and lipid metabolism. Membrane composition and structure are illustrated in panel (**A**). The undissociated form of acetic acid can diffuse passively across the lipid bilayer, while the dissociated form is actively removed from the cell by the transporters Pma1 and Tpo3. Chemical equilibrium exists between the dissociated and undissociated forms of acetic acid both inside and outside the cell, depending on the pH (pKa ~ 4.8). Intracellular concentration is dependent on the equilibrium of undissociated form inside and outside the cell. The plasma membrane is composed of cylindrical (sphingolipids, PC, PS, PI) and conically shaped lipids (with a small head: DAG, PA, PE; or a large head: lyso-glycerophospholipids). A simplified illustration of *S. cerevisiae* lipid metabolism is shown in panel (**B**) (chemical structures: additional file 1, S10). The *Arabidopsis thaliana* genes used in strain engineering (*FAE1*, *GPAT5*) are shown in grey. *AcCOO-* acetate, *AcCOOH* acetic acid, *CDP-DAG* cytidine diacylglycerol, *Cer* ceramides, *CL* cardiolipin, *DAG* diacylglycerol, *EE* ergosterol ester, *ELO* fatty acid elongation, *FAE1*, (*Arabidopsis thaliana*) fatty acid elongase 1; FAS, (de novo) fatty acid synthesis; *GPAT5*, (*Arabidopsis thaliana*) glycerol-3-phosphate acyltransferase 5; *IPC* inositol phosphorylceramide; M(IP)_2_C, mannosyl-di-(inositol phosphoryl) ceramide, *MIPC* mannosyl-inositol phosphorylceramide, *PA* phosphatidic acid, *PC* phosphatidylcholine; *PE* phosphatidylethanolamine, *PG* phosphatidylglycerol; *PI* phosphatidylinositol, *PS* phosphatidylserine, *TAG* triacylglycerol.

Acetic acid is a common inhibitor found in lignocellulosic hydrolysates, and it can enter the cell via several routes, depending on the growth conditions and the physiological state of the cell^5^. In particular, acetic acid can access the intracellular milieu via passive or active transport. The former is relevant for the undissociated form of acetic acid and it includes passive simple diffusion across the lipid bilayer, as well as facilitated diffusion mediated by permeases^6^. In addition, acetate anion is actively transported inside the cell via proton symporters^7^. Finally, ATP-dependent pumps extrude the anion from the cell and are required to compensate for intracellular acetate accumulation. Acetic acid concentration in pre-treated lignocellulosic sugar streams usually varies between 5 and 10 g/L (83–167 mM), although this depends on the raw material and type of pre-treatment^8^. The typical pH of lignocellulosic fermentation media is 4.5–5.5, at which acetic acid uptake occurs predominantly by passive diffusion of the undissociated acetic acid across the plasma membrane (PM) (Fig. 1A)^7^. Once inside the cell, acetic acid can be very damaging to *Saccharomyces cerevisiae* and even trigger apoptosis^9,10^. It is therefore of the utmost importance to minimize the intracellular acetic acid concentration. This can be achieved by decreasing the ingress, increasing the efflux, or metabolising the acetic acid. Acetic acid is not usually metabolised by *S. cerevisiae* in the presence of glucose^11^, and efflux by active transport is energetically costly^12^, and thus, this research focussed on reducing the ingress. Research on the modification of the membrane composition of organisms has recently been summarized in a review by Qi et al.^13^.

The PM of *S. cerevisiae* is composed of glycerophospholipids, sphingolipids, sterols and membrane proteins. The main protein in the PM is Pma1^14^, and the main sterol is ergosterol (ERG). The latter can reside partly in the interior of the lipid bilayer, increasing membrane rigidity^15^. The lipid fraction of the *S. cerevisiae* PM consists of several lipids, and a simplified schematic of *S. cerevisiae* lipid metabolism is shown in Fig. 1B (for chemical structures, see Additional file 1, S1). More comprehensive descriptions of *S. cerevisiae* lipid metabolism have been published previously^16,17^. In *S. cerevisiae*, de novo fatty acid (FA) synthesis is catalysed by a complex consisting of Fas1 and Fas2. This complex produces acyl chains with lengths of up to eighteen carbon atoms (C18). Elongation of fatty acids (FAs) up to C24 is performed by Elo2, while Elo3 is responsible for the elongation of FAs up to C26. The C26 FAs produced by Elo3 are typically used for sphingolipid production. The glycerophospholipids (GPLs) are the main group of membrane lipids, and their important components are included in Fig. 1B (chemical structures: Additional file 1, S1). GPL headgroups with one, rather than two, acyl chains are called lyso-glycerophospholipids (L-GPLs) and are conically shaped due to the large headgroup and small tail end (Fig. 1A). Other membrane lipids also have characteristic shapes: sphingolipids, phosphatidylcholine (PC), phosphatidylserine (PS) and phosphatidylinositol (PI) are considered cylindrical, while phosphatidic acid (PA), phosphatidylethanolamine (PE) and diacylglycerol (DAG) are considered conical with a small headgroup (Fig. 1A). The shape can affect the membrane structure and, in addition to the different membrane components and their headgroups, FA chain length and degree of saturation are important factors affecting membrane properties such as the area per lipid (APL) and the rigidity of the membrane^18,19^.

The properties of the PM determine the rate of uptake of acetic acid as it enters the cell through passive diffusion. Generally, thicker and more rigid membranes impede passive diffusion^20^. We therefore propose membrane engineering as a tool to achieve thicker and more rigid membrane properties, which in turn should reduce acetic acid uptake rate in *S. cerevisiae*. Specifically, we propose the engineering of *S. cerevisiae* to incorporate longer acyl chains into GPLs. In *S. cerevisiae*, very-long-chain FAs are almost exclusively used in the production of storage lipids and sphingolipids. However, *S. cerevisiae* is able to incorporate very-long-chain FAs into GPLs, which occurs for example when sphingolipid production is disrupted^21^. Plants have a range of FA elongases and these are thought not to be affected by *S. cerevisiae* lipid elongation control systems^22^. Some *Arabidopsis thaliana* elongases have previously been expressed in *S. cerevisiae*, each producing a specific FA profile^22^. With respect to the goal of elongating yeast membrane lipids, FAE1 (KCS18) was identified as the most interesting due to its role in the production of FAs with chain lengths of up to C26 (Fig. 1B). Once produced, the FAs can be incorporated into phospholipid biosynthesis, a function performed by glycerol-3-phosphate acyl transferase (GPAT). Plants have a range of GPAT enzymes and each enzyme may have a preference for specific FAs^23^. *A. thaliana* GPAT5 has a preference for FAs with chain lengths compatible with FAE1 production^23^. Therefore, the engineering strategy used to incorporate longer-chain FAs into *S. cerevisiae* GPLs was to overexpress the *A. thaliana* genes *FAE1* and *GPAT5* under strong promotors via integration of the recombinant constructs in the *S. cerevisiae* genome (Fig. 1B).

In order to predict which changes in membrane composition could have the most relevant effect on membrane rigidity and permeability, we modelled the expected outcome of our engineering strategy using Molecular Dynamics (MD) simulations. Shotgun lipidomics were employed for lipidomic profiling of all strains produced in this study and the data was used to analyse membrane composition and provide valuable information and insight into the effects of this engineering approach. Finally, we investigated whether changes in the membrane affected the intracellular concentrations of acetic acid over time.

## Results and discussion

### Very long-chain glycerophospholipids increase the rigidity of in silico membranes in a concentration-dependent manner

MD simulations were used to investigate how increasing the concentrations of very-long-chain FA GPLs affect the structural properties of yeast membrane models. PM permeability in *S. cerevisiae* is dependent on its lipid composition^24,25^. In particular, lipids with long- and very-long-chain FAs determine membrane rigidity by increasing the membrane thickness and lipid packing^19^. Very-long-chain GPLs were introduced into an in silico model of yeast membrane (i.e. Null-256), constructed based on a published model whose structural properties have been previously investigated^24,25^. It consists of 256 lipid molecules: 40 IPC as representative of sphingolipids, 88 DOPC and POPI as representatives of glycerophospholipids, and 40 ergosterol molecules.

**Figure 2:**
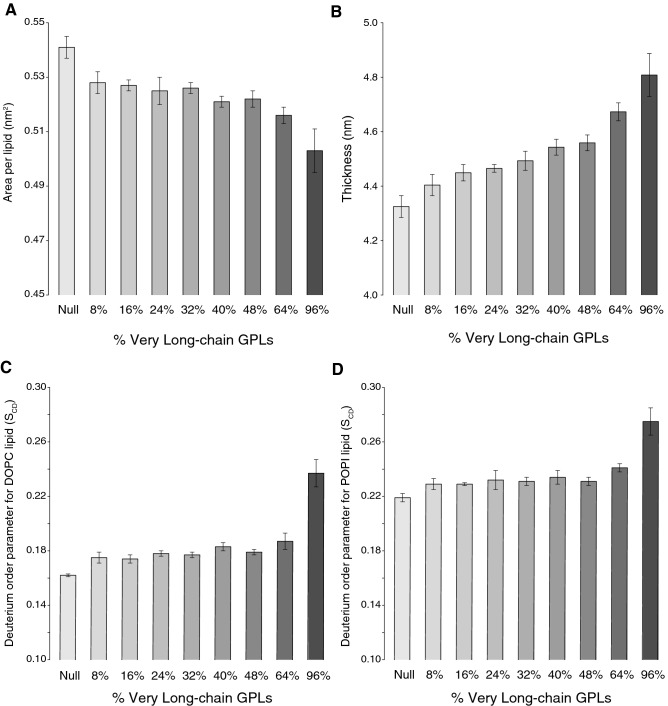
Very-long-chain glycerophospholipids increase the packing and rigidity of yeast membrane models in a concentration-dependent manner. Mean values and associated standard deviations of the structural properties of the membranes investigated in this study: (**A**) the area per lipid (APL), (**B**) the membrane thickness (MT) and (**C**,**D**) the deuterium order parameter (S_CD_) of the *sn1* acyl chains of DOPC and POPI. Simulations were performed for a membrane system with no very-long-chain GPLs (indicated as Null) and for membrane systems with increasing concentrations of very-long-chain GPLs from 8 to 96%. The very-long-chain GPLs were introduced using a 4:2:1 ratio replacing DOPC and POPI molecules The average values were calculated using only three replicates per system to have comparable results. *DOPC* 1,2‐dioleoyl‐sn‐glycerol‐3‐phosphocholine; *GPL* glycerophospholipid, *POPI* 1‐palmitoyl‐2‐oleoyl‐sn‐glycerol‐3‐phospho‐(1′‐myo-inositol).

Starting from the reference system, i.e. Null-256, eight different bilayer membrane were constructed, each having a specific lipid composition (Table 1). The number of sterol and sphingolipid molecules (i.e. ERG and IPC) was constant in all the systems. The long-chain lipids of the PC and PI classes in the reference system (i.e. DOPC (1,2‐dioleoyl‐*sn*‐glycerol‐3‐phosphocholine) and POPI (1‐palmitoyl‐2‐oleoyl‐*sn*‐glycerol‐3‐phospho‐(1′‐myoinositol)) were replaced with very-long-chain GPLs with increasing relative abundances (8, 16, 24, 32, 40, 48, 64 and 96%) (Table 1), rising the average GPL chain length by 0.017 carbon atoms/%_increase_. We used GPLs containing acyl chain lengths of 20 (AOPC or 1-arachidoyl-2-oleoyl-sn-glycero-3-phosphocholine; AOPI or 1-arachidoyl-2-oleoyl-sn-glycero-3-phospho*‐(1'‐myo‐inositol)*), 22 (BOPC or 1-behenoyl-2-oleoyl-sn-glycero-3-phosphocholine; BOPI or 1-behenoyl-2-oleoyl-sn-glycero-3-phospho*‐(1'‐myo‐inositol)*) and 24 (LOPC or 1-lignoceroyl-2-oleoyl-sn-glycero-3-phosphocholine; LOPI or 1-lignoceroyl-2-oleoyl-sn-glycero-3-phospho*‐(1'‐myo‐inositol)*) carbons at the *sn1* position, based on the expected fatty acid production profile of FAE1^22^. We included GPLs with *sn1* chain lengths of C20, C22, and C24 in a constant ratio of 4:2:1, respectively. These ten membrane systems were used as starting points to perform multi-replicate 200–500 ns unbiased MD simulations using a modified version of Stockholm lipids force field (Slipids ff)^24^ (Table 1). The MD ensembles were studied by calculating three parameters: (i) the APL (ii) the membrane thickness (MT) and (iii) the deuterium order parameter (S_CD_) of the *sn1* acyl chains of POPI and DOPC (materials and methods: analysis of membrane properties) (Fig. 2, Additional file 1S3-5-4).

**Table 1:** Lipid compositions of the yeast membrane systems.

Systems	ERG	IPC	DOPC	POPI	AOPC/AOPI	BOPC/BOPI	LOPC/LOPI	Lipids	Ntot	Long chain GPL %	% tot	N° Replicates	Length
NULL 64	10	10	22	22	0	0	0	64	16,192	0%	0%	1	200 ns
NULL 256	40	40	88	88	0	0	0	256	64,768	0%	0%	4	200 ns
M1	40	40	81	81	4	2	1	256	64,956	8%	5%	4	500 ns
M2	40	40	74	74	8	4	2	256	65,144	16%	11%	4	500 ns
M3	40	40	67	67	12	6	3	256	65,332	24%	16%	4	500 ns
M4	40	40	60	60	16	8	4	256	65,520	32%	22%	4	500 ns
M5	40	40	53	53	20	10	5	256	65,708	40%	27%	5	500 ns
M6	40	40	46	46	24	12	6	256	65,896	48%	33%	3	300 ns (2)—500 ns (1)
M7	40	40	32	32	32	16	8	256	66,272	64%	44%	3	300 ns (1)—500 ns (1)
M8	40	40	4	4	48	24	12	256	67,024	96%	66%	3	300 ns (1)—500 ns (2)

Eight lipid membrane systems (represented M1 to M8) with increasing content of different very-long-chain GPLs (8, 16, 24, 32, 40, 48, 64 and 96 mol%) were constructed, starting from two reference yeast membrane models (denoted NULL-64 and NULL-256, see “Materials and methods” section). Long-chain GPLs AOPC and AOPI with the *sn1* acyl chain length of 20 carbons, BOPC and BOPI with the *sn1* acyl chain length of 22 carbons, LOPC and LOPI with the *sn1* acyl chain length of 24 carbons, were introduced at a ratio of 4:2:1, by replacing the representatives of PC and PI lipids (DOPC and POPI, respectively). The same concentrations of sterols and sphingolipids (ERG and IPC, respectively) were maintained in each system as in the reference. The content of very-long-chain GPLs is expressed as a percentage of the total content of GPLs (% GPL, including DOPC, POPI and long-chain GPLs) and the overall number of fatty acid chains (% Tot). The total number of lipid molecules (Lipids) and atoms in each membrane model (N_tot_) are also reported. For most of the systems, up to five replicates (No. of replicates) of MD simulations were collected, each of 200–500 ns length.

*AOPC* 1-arachidoyl-2-oleoyl-sn-glycero-3-phosphocholine, *AOPI* 1-arachidoyl-2-oleoyl-sn-glycero-3-phospho*‐(1'‐myo‐inositol)*, *BOPC* 1-behenoyl-2-oleoyl-sn-glycero-3-phosphocholine, *BOPI* 1-behenoyl-2-oleoyl-sn-glycero-3-phospho*‐(1'‐myo‐inositol)*, *DOPC* 1,2‐dioleoyl‐sn‐glycerol‐3‐phosphocholine, *ERG* ergosterol, *GPL* glycerophospholipid, *IPC* inositol phosphorylceramide, *LOPC* 1-lignoceroyl-2-oleoyl-sn-glycero-3-phosphocholine, *LOPI* 1-lignoceroyl-2-oleoyl-sn-glycero-3-phospho‐(1'‐myo‐inositol), *MD* molecular dynamics, *PC* phosphatidylcholine, *PI* phosphatidylinositol, *POPI* 1‐palmitoyl‐2‐oleoyl‐sn‐glycerol‐3‐phospho‐(1′‐myo-inositol).

A clear trend was observed in the MD simulations, showing that very-long-chain GPLs affected the structural properties of the membrane in a concentration-dependent manner (Fig. 2). Increasing concentrations of very-long-chain lipids resulted in denser (lower APL), thicker (larger MT) and more rigid (higher S_CD_) membranes. The increasing number of very-long-chain GPLs raises the attractive van der Waals forces between acyl chains, leading to an increase in the packing density of the lipid molecules in the membrane, and a gradual decrease in APL (from 0.541 nm^2^ ± 0.004 for the Null system to 0.503 nm^2^ ± 0.008 at 96%) (Fig. 2A). The greater proportion of very long acyl chains in the tightly packed membrane increases the MT (from 4.325 nm ± 0.040 for the Null system to 4.808 nm ± 0.079 at 96%) (Fig. 2B). S_CD_ exhibited a less clear behaviour, and a substantial increase was seen only at 96%; the trend being similar for both the DOPC and POPI *sn*1 acyl chain (Fig. 2C,D). Overall, the results of the MD simulations suggest that very-long-chain GPLs reshape the membrane towards more rigid and more densely packed states in a concentration-dependent manner, thereby reducing their fluidity, which is important in determining their permeability to small molecules such as acetic acid^26^. In addition to these results, it has previously been shown that the GPLs and sphingolipids in the acetic-acid-tolerant *Zygosaccharomyces bailii* not only have longer acyl chains, but this strain is also able to increase the average length of its membrane lipids under acetic acid stress by increasing the relative abundance of sphingolipids^27^. The results of our simulations and the above findings thus indicate that elongating the GPL acyl chain length could reduce acetic acid uptake.

### Heterologous expression of plant *FAE1* and *GPAT5* results in an increase in lipid chain length

Synthetic, codon-optimised versions of *A. thaliana* genes *FAE1* and *GPAT5* (Additional file 1-S6) were inserted into expression vectors using modular cloning for *S. cerevisiae*^28^. Introduction of the *FAE1* and *GPAT5*-containing plasmids into *S. cerevisiae* resulted in two single-gene expression transformants containing a plasmid, and one double transformant with *FAE1* and *GPAT5* integrated into the genome (FAE1_GPAT5) (plasmid maps: Additional file 1-S6). A control strain was produced containing a standard integration vector without gene expression in *S. cerevisiae* (apart from the marker) (plasmid map: Additional file 1-S7). Initial characterisation of the single and double transformants revealed high clonal variation for the single transformants. In addition, the *GPAT5* single transformants showed significantly reduced growth and inconsistent specific growth rate, even when working with a specific clone. It is known that 2-micron-based gene expression can lead to high variation in both plasmid and gene copies^28^. Therefore, experimental findings regarding the single transformants will not be further discussed in this paper. However, information is available in the supplementary material (Additional File 1-S8, S9; Additional file 2). No significant differences were seen in the growth characteristics of the double transformant and those of the control strain; not in the growth profiler set-up (Additional file 1-S10), nor in aerobic shake flask experiments using defined buffered medium (pH 5) (data not shown).

A screening of the aforementioned strains by fatty acid methyl esterification and gas chromatography analysis of total lipids was performed. This revealed that 15% (± 1) of the total lipids in the FAE1_GPAT5 double expression strain had chain lengths of C20-24, while the control strain contained only 0.20% (± 0.07) total lipids with these chain lengths (Additional file 1-S11). Thus, the engineering strategy was successful in increasing the length of FAs in the total lipids in *S. cerevisiae*.

### Shotgun lipidomics confirmed successful strain engineering and revealed a shift in lipid profiles towards increased acyl chain length and more conically shaped lipids

To analyse the impact of the metabolic engineering strategy on the PM more precisely, shotgun lipidomics were performed in triplicate on a representative transformant of each of the different strains. Total cell extracts were used for lipidomics and analysis included all common lipid metabolites and intermediates, including sphingolipids. The analysis also included ergosterol esters (EEs), although free esters (i.e. ERG) could not be included. The raw lipid data is available in the supplementary material (Additional file 2).

The shotgun lipidomics detected a total of 277 and 534 separate metabolites for the control strain and the FAE1_GPAT5 double expression strain, respectively. The control strain exhibited the highest relative abundance of unsaturated lipids. The average number of double bonds per membrane lipid molecule is 1.27 (σ = 0.03) in the FAE1_GPAT5 double expression strain, while this is 1.34 (σ = 0.01) for the control strain. Overall, more saturated membranes are more rigid membranes, as unsaturated lipids increase fluidity and APL^29^. A previous study in *Escherichia coli* have shown that more saturation leads to increased tolerance to carboxylic acids such as octatonic acid^30^.

As our primary interest was the PM, phosphatidylglycerol (PG) and cardiolipin (CL) were considered separately, as they are solely present in mitochondrial membranes. The FAE1_GPAT5 double expression strain had a significantly higher proportion of FAs residing in the PM than the control strain: 71.0% (σ = 1.0) versus 68.0% (σ = 0.5), respectively. The relative abundances of FA chains in different lipid groups are summarised in the supplementary material (Additional file 1-S8).

#### Glycerophospholipids and sphingolipids

The goal of strain engineering in this study was to create thicker, more rigid membranes by elongation of the glycerophospholipids’ fatty acids. Hence, the main goal of the lipidomics was to confirm that the chain elongation revealed in the total lipid screening persisted in PM lipids, including the sphingolipids, as they play a major role in membrane thickness and rigidity.

**Figure 3:**
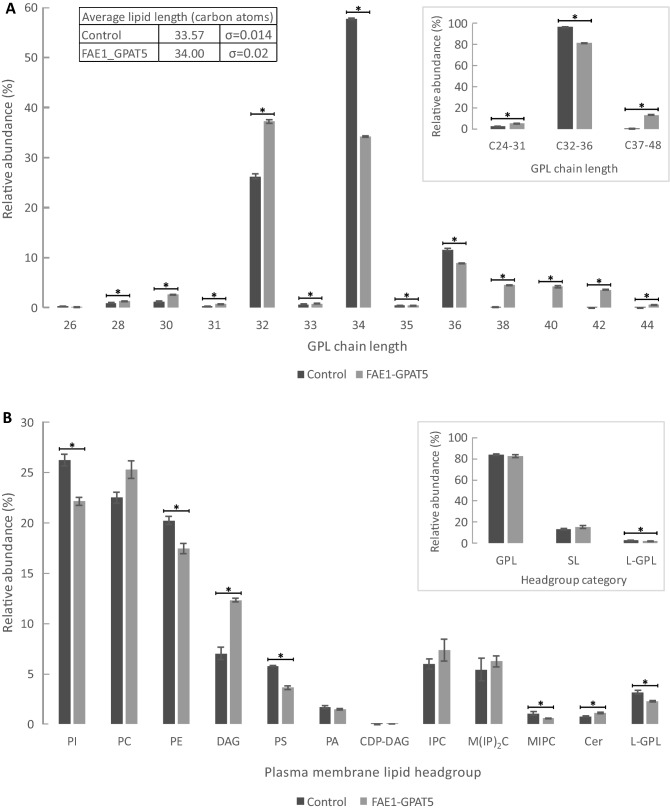
Glycerophospholipid acyl chain length and relative abundance of plasma membrane headgroups. Panel (**A**) shows the combined chain length of the two acyl chains of glycerophospholipids obtained from total lipid analysis for both the control strain (black) and the FAE1_GPAT5 double expression strain (grey). The average carbon length of the FA chains is shown in the table and the cumulative relative amounts of medium- (C24-31), long- (C32-36) and very-long- (C37-48) chain glycerophospholipids are shown in the insert. Panel (**B**) shows the relative abundances of membrane lipid headgroups, and the insert shows the cumulated amounts of different lipid classes. Asterisks indicate significant differences at the 95% confidence level. *CDP-DAG* cytidine diacylglycerol; *Cer* ceramides, *DAG* diacylglycerol, *GPL* glycerophospholipid; *IPC* inositol phosphorylceramide; *L-GPL* lyso-glycerophospholipid, *M(IP)*_*2*_*C* mannosyl-di-(inositol phosphoryl) ceramide; *MIPC* mannosyl-inositol phosphorylceramide; *PA* phosphatidic acid, *PC* phosphatidylcholine; *PE* phosphatidylethanolamine, *PI* phosphatidylinositol, *PS* phosphatidylserine, *SL* sphingolipid, *TAG* triacylglycerol.

A simplified method of comparing complex lipid chain length data is to compare average chain lengths over all the membrane lipids of different strains, and this is also a suitable way of comparing wet-lab results to the MD simulations. It was found that the FAE1_GPAT5 double expression transformant exhibited a clear increase in the average number of carbons per GLP molecule compared to the control strain. More specifically, the FAE1_GPAT5 strain had, on average, 34.00 (σ = 0.02) carbons in the FA chains of a single GPL molecule, while the value for the control strain was only 33.57 (σ = 0.014) carbons per GPL molecule (Fig. 3A). This means that engineering increased the average chain length by about 0.43 carbons per GPL molecule. The MD simulations predicted relatively longer carbon chains on average (34 carbons for Null-256), but the increase in the average GPL chain length from the lipidomic data was comparable to that in the MD system containing 24% very-long-chain GPLs (0.41 increase in carbon chain length) (Table 1). When comparing this MD model to the Null-256 model, the APL decreases from 0.541 nm^2^ (σ = 0.004) to 0.525 nm^2^ (σ = 0.005) (Fig. 2, Additional file 1-S2), and the MT increases from 4.325 nm (σ = 0.04) to 4.465 nm (σ = 0.014) (Additional file 1-S2), suggesting changes expected to increase the membrane rigidity.

The very-long-chain glycerophospholipids were clearly more abundant in the FAE1_GPAT5 double expression strain than in the control strain (Fig. 3A). The fraction of GPL that had < 36 carbons in both fatty acid chains combined was 13.22% (σ = 0.17), while it was only 0.200% (σ = 0.009) in the control strain. Again, this is expected to increase membrane rigidity and MT. Previous studies in yeast have achieved increased C18 FA abundance, increasing ethanol tolerance^31,32^. In *E. coli*, an increase in long chain fatty acids has been achieved in an attempt to decrease the toxicity of free fatty acid production^33^.

In addition to changes in the chain length, changes were also observed in the headgroups (Fig. 3B). The cylindrical membrane lipids (PC, PS, PI and sphingolipids) were the most abundant in both strains. However, cylindrical membrane lipids were slightly less abundant in FAE1_GPAT5 double expression strain (66.5%, σ = 0.6) than in the control strain (67.9% σ = 0.4). Conically shaped membrane lipids are found in two groups: the first being lipids with only one fatty acid chain, where the head is the largest part of the molecule (L-GPL), and the second being lipids with two fatty acid chains, where the headgroup is the smallest part of the molecule (DAG, PA, PE) (Fig. 1A). Lipids with a small headgroup constituted 31.3% (σ = 0.5) of membrane lipids in the FAE1_GPAT5 strain, which was significantly higher than in the control strain (28.99%, σ = 0.18). The most conically shaped membrane lipids, DAGs, were particularly more abundant when *GPAT5* was overexpressed: 12.34% (σ = 0.16) in the FAE1_GPAT5 strain, while this was only 7.0% (σ = 0.6) in the control strain (Fig. 3B). The GPL lipids PS and PI were significantly reduced in the FAE1_GPAT5 double expression strain, compared to the control strain (Fig. 3B). These GPLs are cylindrical, positively charged, and enriched in the inner membrane leaflet. The proportions of conically shaped lipids that enrich in the outer leaflet (sphingolipids and PC) did not differ significantly between the two strains (Fig. 3B). When phosphatidylserine synthase (PssA) was overexpressed in *E. coli*, to increase tolerance to C8 fatty acid production, researchers also noticed an increase in growth rate under acetate stress conditions^34^. In this research, complex differences in relative abundance of different headgroups and increase in average chain length of membrane fatty acids were found.

Changes in headgroups were outside the scope of the modelling systems used in this study, and thus it is impossible to predict how these changes will affect the membrane on a cellular level. However, individual changes can be considered. An increase in cylindrical lipids could lead to more curvature in the membrane (increasing membrane surface) and/or could lead to stresses in the membrane and even disruption of the lamellar structure^15,26,35,36^. Changes in curvature and stress can in turn affect membrane proteins^37,38^. The reduction in PS and PI should result in a lower surface charge of the membrane. It has previously been shown that different proteins localise towards differently charged parts of the membrane and it is hypothesised that charge can provide a cue for protein relocalization^39,40^. It is surprising that both PS and PI are less abundant, as *S. cerevisiae* has been shown to compensate a defect in PS biosynthesis by increasing PI^41^. However, it has been shown that yeast can have massive differences in PI abundance between early and mid-exponential state of cell growth^42^. Despite using the same sampling OD_600_ for all lipid samples in this study, it is possible that at the sampling OD_600_, the double expression strain was closer to mid-exponential growth phase, while the control strain was closer to early exponential growth phase.

#### Storage lipids

The storage lipids analysed were of two groups: the triacylglycerols (TAGs) and the EEs (Fig. 1B). In the FAE1_GPAT5 double expression strain, more very-long-chain FAs were incorporated in both TAGs and EEs than in the control strain. In the control, the TAGs generally had acyl chains of 50 or 52 carbons in total (67.1%, σ = 0.5), while only 9.6% (σ = 0.5) of the TAG total FA chain length was C53-60. The FAE1_GPAT5 strain, in contrast, had longer-chain FAs (C53-60) in 31.8% (σ = 0.8) of the TAGs, while only 39.6% (σ = 1.1) of TAG total FA chain length was C50-52. The EEs of the control strain consisted of 99.1% (σ = 0.2) ergosterol bound to an acyl chain of either C16 or C18, and no EEs with longer FA chains were detected. However, in the FAE1_GPAT5 strain, 3.57% (σ = 0.05) of the EEs were bound to chains longer than C18. Finally, FAE1_GPAT5 exhibited a reduced relative abundance of EEs. It is unclear if there is a correlation between EE abundance and sterol abundance, however, if the sterols would also be less abundant in the double expression strain than the control strain, this could have a significant effect on the membrane. While sterols reduce membrane fluidity and permeability, they also prevent membranes to transition to solid gel states^43^. Thus, a reduction in sterols could significantly increase membrane fluidity and permeability.

It can be summarised that the FAE1_GPAT5 double expression strain has longer FA chains in all different lipid groups in comparison to the control strain. Additionally, many changes in relative abundance of headgroups were observed (Fig. 3B).

### The maximum intracellular acetic acid concentration in the FAE1_GPAT5 strain was reduced, although the uptake rate was higher

As the metabolic engineering strategy was successful in terms of elongating the FAs in membrane lipids, the acetic acid uptake rates of the strains were evaluated. The four concentrations of acetic acid tested (0.56, 2.4, 20 and 144 mM), include low concentrations of acetic acid that should not induce stress, as well as high concentrations, which are more industrially relevant^4^. Experimentally, the low concentrations provide clearer insight in the uptake rate, while high concentrations are more relevant to assess stress conditions. The experiments are performed at pH 5.0 (buffered), which is a typical pH for lignocellulosic fermentation media. It is important to note that acetic acid uptake at pH 5 occurs predominantly by passive diffusion of the undissociated acetic acid across the PM (Figs. 1A, 5)^25^. Hence, acetic acid stress should be considered in terms of the concentration of the undissociated form, rather than the total concentration (pKa ~ 4.8). The intracellular pH of *S. cerevisiae* is generally higher than the pKa of acetic acid, causing the dissociation of acetic acid upon entering the cell (Fig. 1A)^44^. This in turn results in intracellular accumulation of anions and/or a reduction of intracellular pH^45,46^. The ingress of undissociated acetic acid and its intracellular dissociation occurs until an equilibrium is reached between the intracellular and extracellular concentrations of undissociated acetic acid.

**Figure 4:**
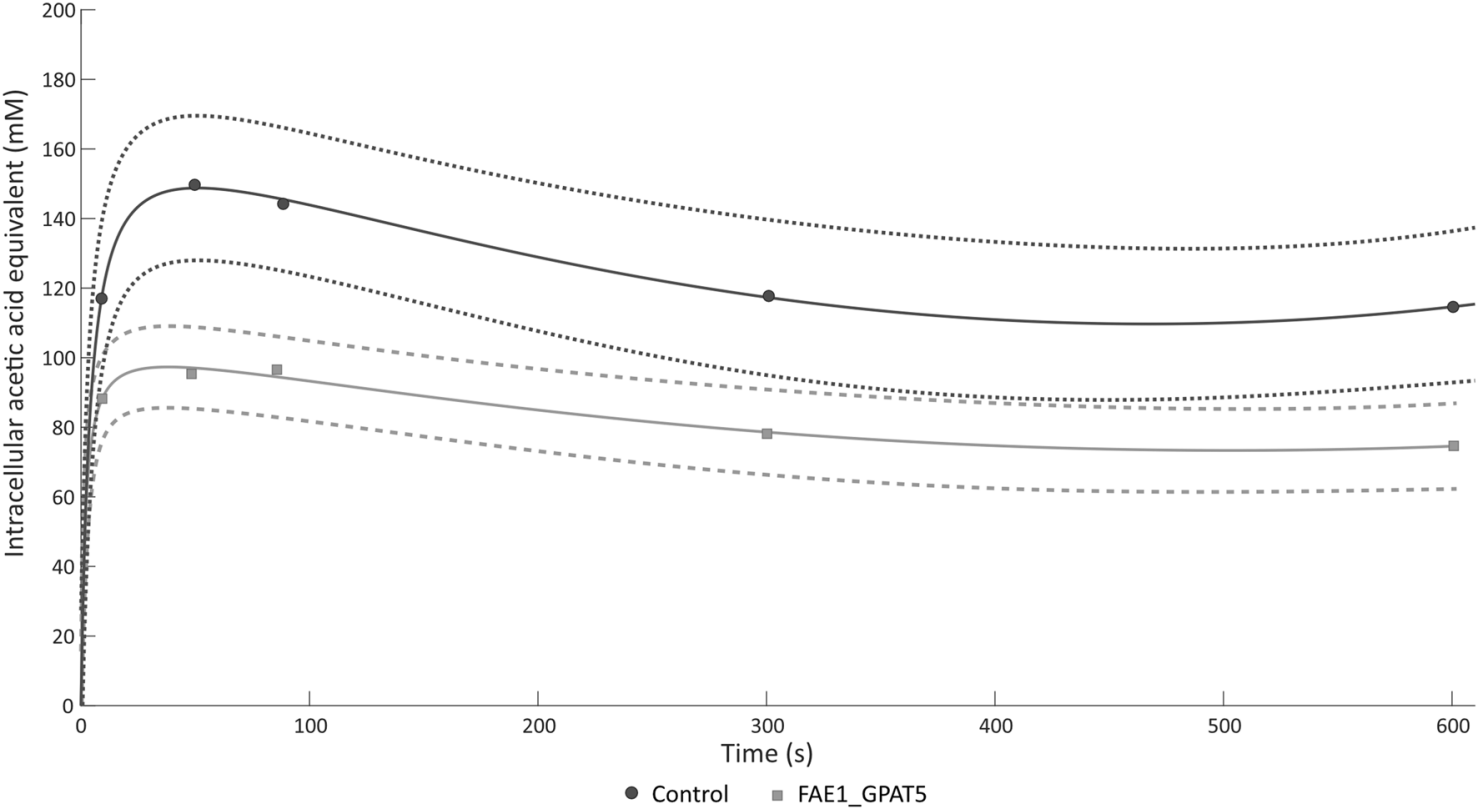
Acetic acid uptake with an initial extracellular acetic acid concentration of 144 mM, pH 5.0. Rational regression lines were calculated using MATLAB and the 90% confidence intervals are shown by the dotted lines. The average sample response is given at each time point measured (10, 45, 80, 300 and 600 s). The control strain is depicted in black, while the FAE1_GPAT5 double expression strain is shown in grey.

The FAE1_GPAT5 double expression strain showed lower maximum intracellular acetic acid concentrations than the control strain at all concentrations investigated (90% confidence level) (Fig. 4, Additional file 1-S12). Incubation at the high, industrially relevant concentration of 144 mM extracellular acetic acid, caused the control strain to rapidly reach a similar intracellular concentration of acetic acid, as can be seen in Fig. 4. The average maximum intracellular concentration of acetic acid in the double expression strain was 96.6 mM, which indicates a more significant drop in intracellular pH compared to the control strain (4.7 versus 5 in the control strain, calculated using the Henderson-Hasselbalch equation). This indicates that the FAE1_GPAT5 double expression strain is more susceptible to acetic acid stress compared to the control strain. This could be due to a range of reasons, including, for example, reduced intracellular buffering capacity, or reduced efflux rate.

These results, together with shotgun lipidomic data, show complex changes in the engineered strain that we did not include in the MD simulations by which we focus on investigate how increasing concentrations of very-long-chain FA GPLs affect the structural properties of models of yeast membranes. Full-atom MD simulations together with full atomistic force fields is a powerful and widely used approach for study lipid membranes, providing important insights on their properties^47,48^. However, this method presents a few shortcomings that does not allow yet to fully reproducing the complexity of lipid profiles collected by lipidomics. Full-atom simulations require a large amount of computer power to investigate lipid membranes, limiting the sampling and spatiotemporal range (e.g., complexity and number of lipid components, dynamics at different timescales, etc.) that can be reached^49^. Simulations of simple bilayers presented in this study composed of a few hundreds of different lipids need to be conducted for at least hundreds of nanoseconds to reach the stabilization of properties as APL, MT, and S_CD_ (Fig. 2 and Table 1). Several methods permit to more effectively sample the phase space, as different biasing and enhanced sampling approaches but with increased computational costs and only recently they are starting to be applied in membrane simulations^49^. Another limitation of the atomistic simulations is the quality of the force field chosen to describe the system. We employed the robust Slipids force field that includes several classes of lipids and provides protocols for adding new lipids, which was previously used for the very-long-chain GPLs^24^. However, the parameters in atomistic force fields are not reliably transferable between different lipids, so developing new ones requires accurate parameterization against experimental structural data. This process is highly challenging since the lipid diversity is enormous and detailed experimental data are available for a limited subgroup of all lipids and largely missing for complex mixtures of them. These limitations do not allow us to fully reproduce by MD simulations the lipid profiles collected by shotgun lipidomics on the control strain and FAE1_GPAT5 strain.

**Figure 5:**
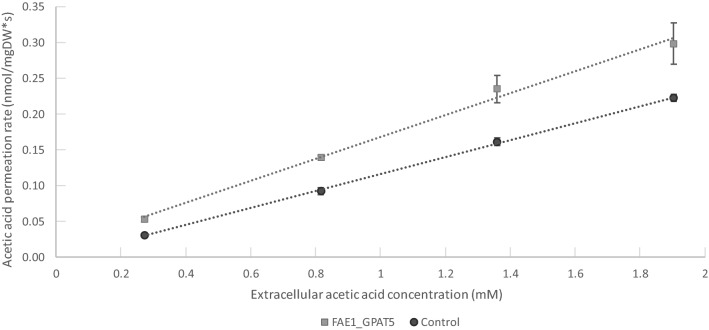
Acetic acid uptake kinetics. The FAE_GPAT5 strain (light grey) and the control strain (dark grey) are shown. Additionally, a linear regression line is shown. The slope of the regression lines is as followed: for FAE1_GPAT5 0.15 (σ 0.02) and 0.118 (σ 0.002) for the control strain. A linear correlation between the extracellular acetic acid concentration (mM, x-axis) and the acetic acid permeation rate (nmol/(mg dry weight x s), y-axis) would suggest that acetic acid uptake occurs predominantly as passive diffusion. The slope of a linear correlation curve is indicative of the net uptake rate.

In order to confirm that the ingress of acetic acid into the cells occurred predominantly by passive diffusion, kinetic experiments were performed on the FAE1_GPAT5 strain and the control strain. The results presented in Fig. 5 show a strong linear correlation (R^2^ ≤ 0.99) between sampling points of the same strain, confirming that the passive uptake of acetic acid is the main method of ingress into the cell. In addition to the linearity of the correlation, the slope can be used to indicate the net uptake rate of acetic acid. The linear regression line for the FAE1_GPAT5 had a slope of 0.15 (σ 0.02), while that for the control strain was 0.118 (σ 0.002), indicating that the net ingress of acetic acid into the FAE1_GPAT5 cells was higher than in the control strain (at the 90% confidence interval). This was in line with the acetic acid uptake measurements, yet in contrast with the original hypothesis that the successful increase of the FA chain length should have resulted in the membrane being thicker and more rigid, which in turn should to reduce passive uptake. However, as mentioned before, the modelling employed in this study only accounted for increasing membrane lipid chain length and the lipidomics showed that, besides longer-chain fatty acids being included in all lipid groups, the headgroups of the GPLs drastically changed. In particular, the FAE1_GPAT5 strain contained significantly higher relative amounts of conically shaped DAGs (Fig. 3B). Previously published results of membrane simulations show that high levels of DAGs are able to break the lamellar structure of the plasma membrane, reducing its integrity^35^. Other research has shown that DAGs can be highly localised in specific areas of the membrane during cellular growth^50^, potentially increasing the damage to the lamellar structure of the membrane. However, low concentrations of DAGs can replace ERG in membrane microdomains^51^, making the membrane more rigid^15^. Besides the membrane structure itself, it is possible that transformants have a different rate of acetic acid efflux, which could influence the net influx of acetic acid.

## Conclusions

The results of the MD simulations indicated that very-long-chain GPLs alter the plasma membrane towards more rigid and packed states in a concentration-dependent manner. Strain engineering was successful in increasing the average FA chain length in all membrane and storage lipids. Despite this, the FAE1_GPAT5 double transformant seemed to have an increased rate of net acetic acid uptake and reduced ability to maintain pH homeostasis compared with the background strain. We suggest that changes in the relative abundance of certain membrane lipid headgroups (e.g. DAG lipids) could mitigate the effect of longer fatty acid chains, resulting in a higher net uptake rate of acetic acid.

## Methods

### Molecular dynamic simulations

#### Membrane modelling and simulation set-up

Eight models of lipid membranes with increasing concentrations of different types of very-long-chain GPLs (indicated as M1-M8, see Table 1) were constructed based on previously published yeast membrane models^24,25^. Multi-replicate unbiased MD simulations were performed for ten systems (Table 1). *Gromacs v5.1*^52^ was used together with a modified version of the Slipids ff^53–55^ in which the topologies and parameters for additional lipids have been included as explained in the original article^24^. Each of the ten system was solvated in a rectangular box of water molecules (40 water molecules for each lipid in the membrane) using the TIP3P model^56^. The net charge was neutralized by adding 32–128 sodium ions depending on the system. The same approach was used for the solvation and charge neutralization of all the membrane systems described below. A membrane system composed of 64 lipid molecules was produced with the CHARMM Membrane Builder^57^. The lipid composition of the membrane was: 10 IPC as representative of sphingolipids, 22 molecules of DOPC as representatives of the PC class, 22 molecules of POPI as representatives of the PI class, and ten molecules of ERG as representatives of the sterols. The resulting system was minimized using a 1000-step steepest-descent algorithm. After minimization, ten random POPI molecules were replaced by ten IPC molecules using an *in-house* Python script (obtaining a system denoted Null-64 in Table 1). A 1000-step minimization was performed followed by an equilibration step of 5 ns in the NPT ensemble with a 4-fs time step. The membrane systems described below followed the same protocol. The equilibrated 64-lipid membrane was then expanded along the x and y dimensions, producing a system composed of 256 lipids. We used this system (denoted Null-256 in Table 1) as the starting point for our study to introduce the very-long-chain GPLs: AOPC and AOPI with the *sn1* acyl chain length of 20 carbons, BOPC and BOPI with the *sn1* acyl chain length of 22 carbons, LOPC, and LOPI with the *sn1* acyl chain length of 24 carbons (Table 1). The AOPC/AOPI, BOPC/BOPI and LOPC/LOPI were added at a ratio of 4:2:1, with total contents of very-long-chain GPLs of 8, 16, 24, 32, 40, 48, 64 and 96% (Table 1). These percentages were calculated as the content of very-long-chain GPLs divided by the total amount of GPLs (i.e., DOPC, POPI, and very-long-chain lipids). The Slipids ff topologies of the very-long-chain GPLs AOPC, BOPC, LOPC and AOPI, BOPI, LOPI were obtained by adapting the topologies of the 1-stearoyl-2-oleoyl-sn-glycero-3-phosphocholine and of the POPI, respectively. This adapting step was performed using *in-house* Python scripts. The *sn1* chains of random DOPC and POPI molecules of the Null-256 system were elongated and saturated. All covalent bonds were constrained with the LINCS algorithm^58^, and periodic boundary conditions were employed for the preparation steps and the productive MD simulations. The Parrinello-Rahman barostat^59^ was used with a 10-ps coupling constant, while the temperature was kept at 298 K using the Nosé-Hoover thermostat^60^ with a 0.5 ps time constant. The particle-mesh Ewald switch summation method was employed to treat electrostatic interactions^61^. Switch cut-offs of 1.2 nm were used for Van der Waals and short-range Coulomb interactions. Three to five replicates of unbiased MD simulations were performed for each of the ten systems, each being of 200–500 ns of length (Table 1). An *in-house* Python script was used to implement the hydrogen mass repartitioning approach^62^ to remove the fastest degrees of freedom in the system, allowing the use time steps up to 4 fs in the MD simulations. The hydrogen mass was increased by a factor of four, from 1.008 u to 4.0320 u. This approach has been tested in simulations of a variety of membranes, showing practically no differences in their structural properties when compared to non-heavy hydrogen simulations^62^. At least three independent replicates were collected for each system (Table 1), using different initial random seeds for the simulation^63,64^.

#### Analysis of the membrane properties

The output of the MD simulations provided three structural parameters commonly used to study membranes and to validate simulation ensembles against experimental data^65^: (i) the APL, (ii) the MT and (iii) the S_CD_. All the analyses were carried out using *in-house* Python scripts.

The APL was calculated as the lateral area of the membrane divided by the total number of lipids in one leaflet:

1 $$APL = \langle L_{x} L_{y} /N\rangle$$

where, *Lx* and *L*y are the lateral dimensions of the membrane, and *N* is the total number of lipids in one leaflet. The angular brackets denote the ensemble average. The average bilayer thickness was calculated as the average distance between the phosphate groups of the lipid heads in two opposing leaflets of the membrane.

The carbon-deuterium (or carbon-hydrogen) order parameter (S_CD_) provides information on the global motional order of the lipid bilayer and details on the motions of the atoms in the acyl chains of lipids^66^. Experimental investigations, as quadrupolar splitting measurements from deuterium NMR experiments, permit to accurately calculate S_CD_^66–69^ and obtain information on dynamics relevant in the time scales accessible by MD simulations. S_CD_ can be calculated from simulations of lipid bilayers, making it an important structural property commonly used for comparison and validation against experimental data^70^. S_CD_ was calculated as a measure of the carbon bond rigidity of the acyl chains in the lipids in the membrane, according to:

2 $$S_{CD} = \frac{1}{2}\langle 3cos^{2 } \theta_{\left( t \right)} - 1 \rangle$$

where θ describes the orientation of the carbon–hydrogen bond vector with respect to the membrane-normal vector at time t and the angle brackets denote an ensemble average over the sampled conformations. S_CD_ was calculated for all the carbon atoms in the *sn1* acyl chains of POPI and DOPC. The three structural parameters were computed for each of the four 200 ns replicates of the Null-256 system and each of the three to five 300 ns replicates of the longer-chain membrane systems. Mean values averaged over the number of simulation frames and standard deviations of the structural parameters were calculated (Additional file 1-S2). The means and standard deviations of the three structural parameters were calculated for three replicates of each membrane system to provide MD ensembles with comparable sizes (Fig. 2 and Additional file 1-S2). Some of the replicates of the membrane systems with very-long-chain GPLs were elongated to 500 ns, confirming that 200–300 ns were sufficient time windows to reach the stabilization of the properties of interest (Table 1, Additional file 1-S4).

### Strains and cultivation media

*Escherichia coli* strain NEB5α (C2987H, New England Biolabs) was used for plasmid propagation and safekeeping. The strain was grown on Lysogeny Broth medium (10 g/L peptone, 5 g/L yeast extract, 5 g/L NaCl with 15 g/L agar). In transformation reactions, the medium was appropriately supplemented with antibiotics (100 µg/mL ampicillin, 25 µg/mL chloramphenicol, 50 µg/mL kanamycin or G418).

*S. cerevisiae* strain CEN.PK113_5D^71^ was grown on complex medium (YPD medium, 20 g/L peptone, 10 g/L yeast extract, 20 g/L glucose, 15 g/L agar) prior to genetic modification. Transformants were selected on synthetic dropout medium (20 g/L glucose, 5 g/L (NH_4_)_2_SO_4_, 1.7 g/L yeast nitrogen base, 0.77 g/L nutritional supplement mixture minus uracil (FORMEDIUM, Norfolk, UK), 20 g/L agar) and transformants were subsequently grown on defined medium^72^ adjusted to pH 5 using potassium hydroxide and buffered using 50 mM potassium hydrogen phthalate.

### Plasmid construction

Synthetic, codon-optimised versions of FAE1 and GPAT5 were obtained (TWIST Bioscience, CA, USA) and integrated into plasmids using modular cloning for *S. cerevisiae*^28^ (Addgene kit #1000000061). Single gene expression plasmids were expressed in *S. cerevisiae* as plasmid. Multigene expression vectors and the “empty vector” control (plasmid map: additional file 1-S7) were integrated into the genome using site-directed integration into the *ura*3-52 mutation of *S. cerevisiae* strain CEN.PK113_5D whilst creating prototrophic transformants. Transformation and integrated gene sequences were verified by PCR and sanger sequencing. Plasmid maps as well as codon optimised gene sequences are available in the Supplementary Material (Additional file 1-S6 and S7).

### *Saccharomyces cerevisiae* transformation

*Saccharomyces cerevisiae* transformation was performed using the PEG-mediated LiOAc method based on Gietz and Woods^73^. No cell titer was used, as instead an initial OD_600_ of 0.4 was used and cells were harvested at OD_600_ 1.0–1.5. Harvested and washed cells were resuspended in 1 mL of 0.1 M LiOAc. For each transformation, 100 µL was transferred into a microcentrifuge tube and centrifuged at 6000 G for 30 s after which the supernatant was removed. The reactions were placed on ice and the following was added in order to each reaction: 240 µL 50% PEG4000, 35 µL 1 M LiOAc, 75 µL DNA mix containing the transformation plasmid (< 5 µg) and 50 µg of single stranded salmon sperm DNA.

### Lipid screening

The relative chain length of the transformants’ total lipids was preliminarily assessed using standard fatty acid methyl esterification and gas chromatography, to analyse acyl chains lengths of C6-24, as previously described^74^.

### Lipidomics

#### Sample preparation

A single colony of *S. cerevisiae* was taken from < 3-day-old defined medium plates and used to inoculate a 50 mL tube containing 15 mL defined medium for overnight incubation at 30 °C, and 200 rpm. A 500 mL baffled shake flask containing 50 mL defined medium was inoculated to an OD_600_ of 0.05, and subsequently incubated at 30 °C, and 160 rpm until an OD_600_ of 0.8 (± 0.03) was reached. The flask was placed in an ice water bath for about 15 min. After cooling, 24 mL of the culture was transferred to a separate 50 mL conical tube and centrifuged (4000 G, 5 min, 4 °C). The pellet was flash-frozen in liquid nitrogen prior to storage at − 80° C. The analysis was performed in triplicate for each strain.

Pellets were resuspended in 1 mL sterile Milli Q water and transferred to fresh Fastprep tubes (0.5 mm zirconium beads, Precellys®, Bertin Instruments) on ice, and cell lysis was performed using a Precellys® Evolution cell disruptor (Bertin Instruments) with five cycles of 20 s, at 6800 rpm. The Fastprep tubes were incubated on ice for 1 min between each cycle. After cell disruption, the samples were centrifuged at maximum speed for 1 min. Following this, 0.5 mL was transferred to fresh microcentrifuge tubes, flash-frozen and stored at − 80 °C. All samples were transported on dry ice to Lipotype GmbH (Tatzberg, Germany) where shotgun lipidomics were performed on total lipids, according to the premium analysis package.

#### Lipid extraction for mass spectrometry lipidomics

Mass spectrometry-based lipid analysis was performed by Lipotype GmbH as described previously^17,42^. Lipids were extracted using a two-step chloroform/methanol procedure^17^. Samples were spiked with an internal lipid standard mixture containing: cytidine diphosphate diacylglycerol (CDP-DAG) 17:0/18:1, Cer 18:1;2/17:0, DAG 17:0/17:0, lysophosphatidate 17:0 (LPA), lyso-phosphatidylcholine 12:0 (LPC), lysophosphatidylethanolamine 17:1 (LPE), lyso-phosphatidylinositol 17:1 (LPI), lysophosphatidylserine 17:1 (LPS), PA 17:0/14:1, PC 17:0/14:1, PE 17:0/14:1, PG 17:0/14:1, PI 17:0/14:1, PS 17:0/14:1, EE 13:0, TAG 17:0/17:0/17:0, stigmastatrienol, IPC 44:0;2, MIPC 44:0;2 and M(IP)_2_C 44:0;2. After extraction, the organic phase was transferred to an infusion plate and dried in a speed vacuum concentrator. First-step dry extract was re-suspended in 7.5 mM ammonium acetate in chloroform/methanol/propanol (1:2:4, V:V:V) and second-step dry extract in a 33% ethanol solution of methylamine in chloroform/methanol (0.003:5:1; V:V:V). All liquid handling steps were performed using a Hamilton Robotics STARlet robotic platform with the Anti Droplet Control feature for organic solvent pipetting.

#### Mass Spectrometer data acquisition

Samples were analysed by direct infusion on a QExactive mass spectrometer (MS) (Thermo Scientific) equipped with a TriVersa NanoMate ion source (Advion Biosciences). Samples were analysed in both positive and negative ion modes with a resolution of R_m/z=200_ = 280 000 for MS and R_m/z=200_ = 17 500 for MS/MS experiments, in a single acquisition. MS/MS was triggered by an inclusion list encompassing corresponding MS mass ranges scanned in 1 Da increments^75^. Both MS and MS/MS data were combined to monitor EE, DAG and TAG ions as ammonium adducts, PC as an acetate adduct, and CL, PA, PE, PG, PI and PS as deprotonated anions. MS only was used to monitor LPA, LPE, LPI, LPS, IPC, MIPC and M(IP)_2_C as deprotonated anions, and Cer and LPC as acetate adducts.

#### Data analysis and post-processing

Data were analysed with in-house developed lipid identification software based on LipidXplorer^76,77^. Data post-processing and normalization were performed using an in-house developed data management system. Only lipid identifications with a signal-to-noise ratio > 5, and a signal intensity fivefold higher than in corresponding blank samples were considered for further data analysis. All analysis was performed using lipid data normalised to protein concentration.

### Acetic acid uptake and kinetic measurements

#### Cell cultivation

A single colony of *S. cerevisiae* was taken from < 3-day-old defined medium plates and used to inoculate a 50 mL tube containing 10 mL defined medium for overnight incubation at 30 °C, 200 rpm). A 1-l baffled shake flask containing 0.1 l defined medium was inoculated to an OD_600_ of 0.05. Flasks were incubated at 30 °C, 160 rpm until the OD_600_ reached 0.8 (± 0.03). Thereafter, 90 mL of cells was harvested and centrifuged at 4 °C, 4000×*g*, for 5 min. The cells were washed twice using ice-cold 50 mM potassium hydrogen phthalate buffer (pH 5.0). The final pellet was resuspended in 800 µL ice-cold potassium hydrogen phthalate buffer (50 mM, pH 5.0) and stored on ice until analysis (< 3 h).

#### Determination of acetic acid diffusion rate and kinetics

The acetic acid diffusion rate was measured as described previously^25^, but using total acetic acid concentrations of 0.56, 2.4, 20 and 144 mM. MATLAB was used to create rational regression lines and confidence intervals.

Next, acetic acid diffusion kinetics were determined as described before^25^. The kinetics experiment used a final amount of 7.4–51.8 kBq (0.148–1.036 Bq/µL) [1-^14^C] acetic acid mixed with 0.2–1.4 mM non-radiolabelled acetic acid, resulting in total acetic acid concentrations of 0.27–1.9 mM, with a specific activity of 39,300 DPM/nmol. Each assay was initiated by incubating 10 µL of cells stored on ice with 30 µL of 50 mM potassium hydrogen phthalate buffer at pH 5.0, in a 30° C water bath for 4 min. Acetic acid diffusion was then measured after the addition of 10 µL acetic acid mixture by incubation at 30֯ C for 30 s. At this time, the assay was terminated by addition of 10 mL ice-cold 2 mM acetic acid stop solution. The cells were swiftly filtered and handled as described above. The wash solution used was ice-cold 2 mM acetic acid.

#### Analysis of intracellular acetic acid concentration

The amount of intracellular acetic acid was determined by measuring the radioactive decay of [^14^C] acetic acid using a liquid scintillation counter (Wallac Guardian 1414, Perkin Elmer). Background radiation was measured in cultures, scintillation liquid and the filters used in a representative way. None of the background controls showed significant amounts of radioactivity, and the average background was subtracted from the samples. The radioactive decay measured was within the linear concentration range and no quenching effects of the sample matrix were observed.

### Cultivation of *Saccharomyces cerevisiae* in a growth profiler

*S. cerevisiae* inoculum was prepared in a 96-well plate (Tissue Culture Testplate®, SPL Life Sciences) containing 150 µL defined medium at pH 5. The culture was grown overnight in a thermomixer (Eppendorf ThermoTop®) at 30 °C, 200 rpm. Cells were resuspended by pipetting prior to measurement of the cell density using a plate reader (Spectrostar Nano®, BMG Labtech). The cells were then transferred to a fresh 96-well plate (CR1496e®, System Duetz) with a final volume of 200 µL and starting OD_600_-equivalent of 0.02. Strain analysis was performed in triplicate. The plate was sealed with a machine-specific aerobic sandwich cover (CR1296a EnzyScreen B.V.). The plate was incubated in a growth profiler (GP960 REV2®, EnzyScreen) for 72 h at 30 °C, 230 rpm, and green light scattering measurements were taken hourly. Defined medium was used as a blank, and the value of the green light scattering signal (green value, GV) obtained from this was subtracted from the values measured during cell growth.

Green values were converted into OD_600_ values using a standard curve obtained previously using *S. cerevisiae* strain CENPK_113.7D^71^ grown in defined medium. The following two equations were used to convert GV into OD_600_ values:

For GV ≤ 10:

3 $$OD600 = 0.0322 x 2.72^{{\left( {0.4328 x GV} \right)}}$$

For GV ≥ 10:

4 $$OD600 = - 0.0004 x GV^{3} + 0.0398 x GV^{2} - 0.6506 x GV + 5.4063$$

## Supplementary Information

Supplementary Information 1.

Supplementary Information 2.

## Data Availability

All the scripts, inputs and outputs, and the documentation required to reproduce the molecular dynamics simulations and analyses used in this article are available in the GitHub repository: https://github.com/ELELAB/YEAST_MEMBRANE_MD. The MD trajectories analysed during this study are available as OSF repository at https://osf.io/3j2ap/. The raw lipid data obtained through Lipotype GmbH is included in this published article as comma delimited file (Additional file 2). Supplementary data required to reproduce the work presented or supporting the conclusions of the current study are included in this published article (Additional file 1).

## Abbreviations

AOPC: 1-Arachidoyl-2-oleoyl-sn-glycero-3-phosphocholine
AOPI: 1-Arachidoyl-2-oleoyl-sn-glycero-3-phospho‐(1'‐myo‐inositol)
APL: Area per lipid
BOPC: 1-Behenoyl-2-oleoyl-sn-glycero-3-phosphocholine
BOPI: 1-Behenoyl-2-oleoyl-sn-glycero-3-phospho‐(1'‐myo‐inositol)
CDP-DAG: Cytidine diphosphate diacylglycerol
Cer: Ceramide
CL: Cardiolipin
DAG: Diacylglycerol
DOPC: 1,2‐Dioleoyl‐sn‐glycerol‐3‐phosphocholine
EE: Ergosterol ester
ERG: Ergosterol
FA: Fatty acid
GPAT: Glycerol-3-phosphate acyl transferase
GPL: Glycerophospholipid
GV: Green value
IPC: Inositol phosphorylceramide
L-GPL: Lyso-glycerophospholipid
LOPC: 1-Lignoceroyl-2-oleoyl-sn-glycero-3-phosphocholine
LOPI: 1-Lignoceroyl-2-oleoyl-sn-glycero-3-phospho‐(1'‐myo‐inositol)
LPA: Lysophosphatidate
LPC: Lyso-phosphatidylcholine
LPE: Lysophosphatidyl­ethanolamine
LPI: Lyso-phosphatidylinositol
LPS: Lysophosphatidylserine
M(IP)_2_C: Mannosyl-di-(inositol phosphoryl) ceramide
MD: Molecular dynamics
MIPC: Mannosyl-inositol phosphorylceramide
MS: Mass spectrometer
MT: Membrane thickness
PA: Phosphatidic acid
PC: Phosphatidylcholine
PE: Phosphatidyl­ethanolamine
PG: Phosphatidylglycerol
PI: Phosphatidylinositol
PM: Plasma membrane
POPI: 1‐Palmitoyl‐2‐oleoyl‐sn‐glycerol‐3‐phospho‐(1′‐myo-inositol)
PS: Phosphatidylserine
S_CD_: Deuterium order parameter
Slipds ff: Stockholm lipids force field
TAG: Triacylglycerol
