# Supplementary material for: Molecular-dynamics-simulation-guided membrane engineering allows the increase of membrane fatty acid chain length in Saccharomyces cerevisiae
Source: Sci Rep. 2021 Aug 30;11:17333. doi: 10.1038/s41598-021-96757-y (PMC8405694; doi:10.1038/s41598-021-96757-y)

Molecular-dynamics-simulation-guided membrane engineering of *Saccharomyces cerevisiae* by expression of plant *FAE1* and *GPAT5* genes increases fatty acid chain length in membrane lipids

Jeroen M. Maertens^a^; Simone Scrima^b^; Matteo Lambrughi^b^; Samuel Genheden^c^, Cecilia Trivellin^a^; Leif A. Eriksson^c^; Elena Papaleo^b^; Lisbeth Olsson^a^; Maurizio Bettiga*^a^

^a^ Department of Biology and Biological Engineering, Division of Industrial Biotechnology, Chalmers University of Technology, Kemivägen 10, SE-412 96 Gothenburg, Sweden

^b^ Computational Biology Laboratory, Danish Cancer Society Research Center, Strandboulevarden 49, 2100 Copenhagen, Denmark

^c^ Department for Chemistry and Molecular Biology, University of Gothenburg, Medicinaregatan 9c 40530 Gothenburg, Sweden

*Corresponding author: Maurizio Bettiga; maurizio.bettiga@chalmers.se

**Table of contents**

S1 – Lipids and chemical structures 3

S2 – Summary of the membrane properties under investigation 4

S3 – Membrane properties as averages (shorter simulations) 5

S4 – Membrane properties as averages (longer simulations) 6

S5 – Boxplot of parameters for membrane simulations 7

S6 – Codon optimised gene sequences 8

S7 – Plasmid maps 9

S8 – Relative abundances lipids 13

S9 – Single expression strains 13

S10 - Growth profiler 17

S11 - Screening lipid data 19

S12 - Additional data for uptake measurements 20

# S1 Lipids and chemical structures

Acetyl-CoA

Acyl-CoA

EE

PA

DAG

PC

CDP-DAG

PI

PG

CL

TAG

Cer

IPC

MIPC

M(IP)2C

PS

PE

R denotes the lipid tail.

**S2 Summary of the membrane properties under investigation**

We reported the average values and associated standard deviations obtained using three 200-300ns replicates for each system.


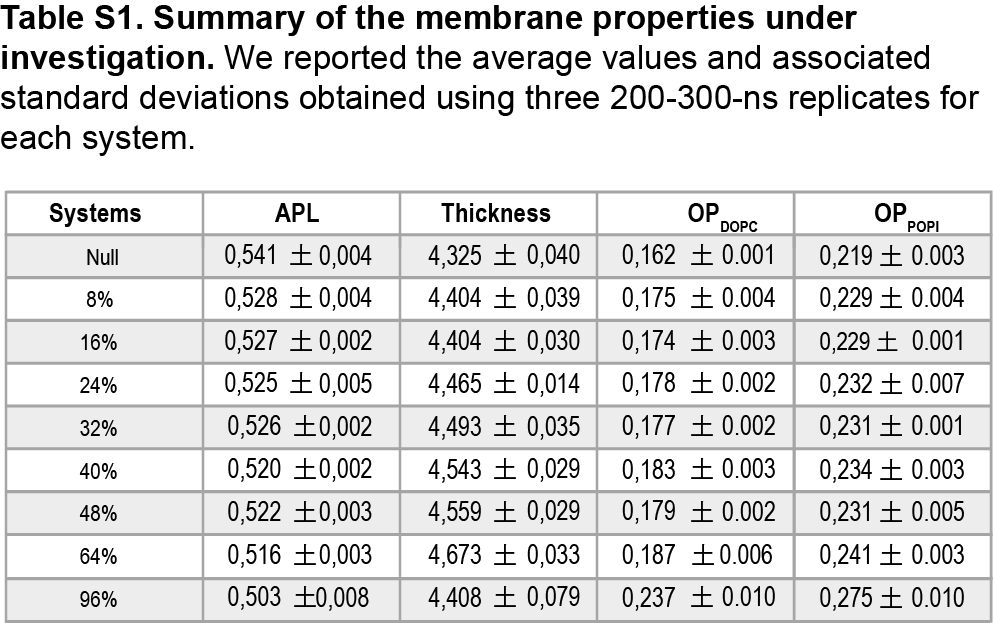


**S3** **Membrane properties as averages (and associated standard deviations) over the 200ns MD replicates of the Null-256 model (referred as Null) and each of the 300-ns replicates of the eight systems with elongated acyl chains.**

The same properties analysed in figure 2 of the main text are reported here for each individual replicate of 200-300ns of simulation length, i.e. (A) Area per lipid (APL), (B) membrane thickness (MT), (C) Deuterium Order parameter for sn-1 of DOPC lipids, (D) Deuterium Order parameter for POPI lipids.


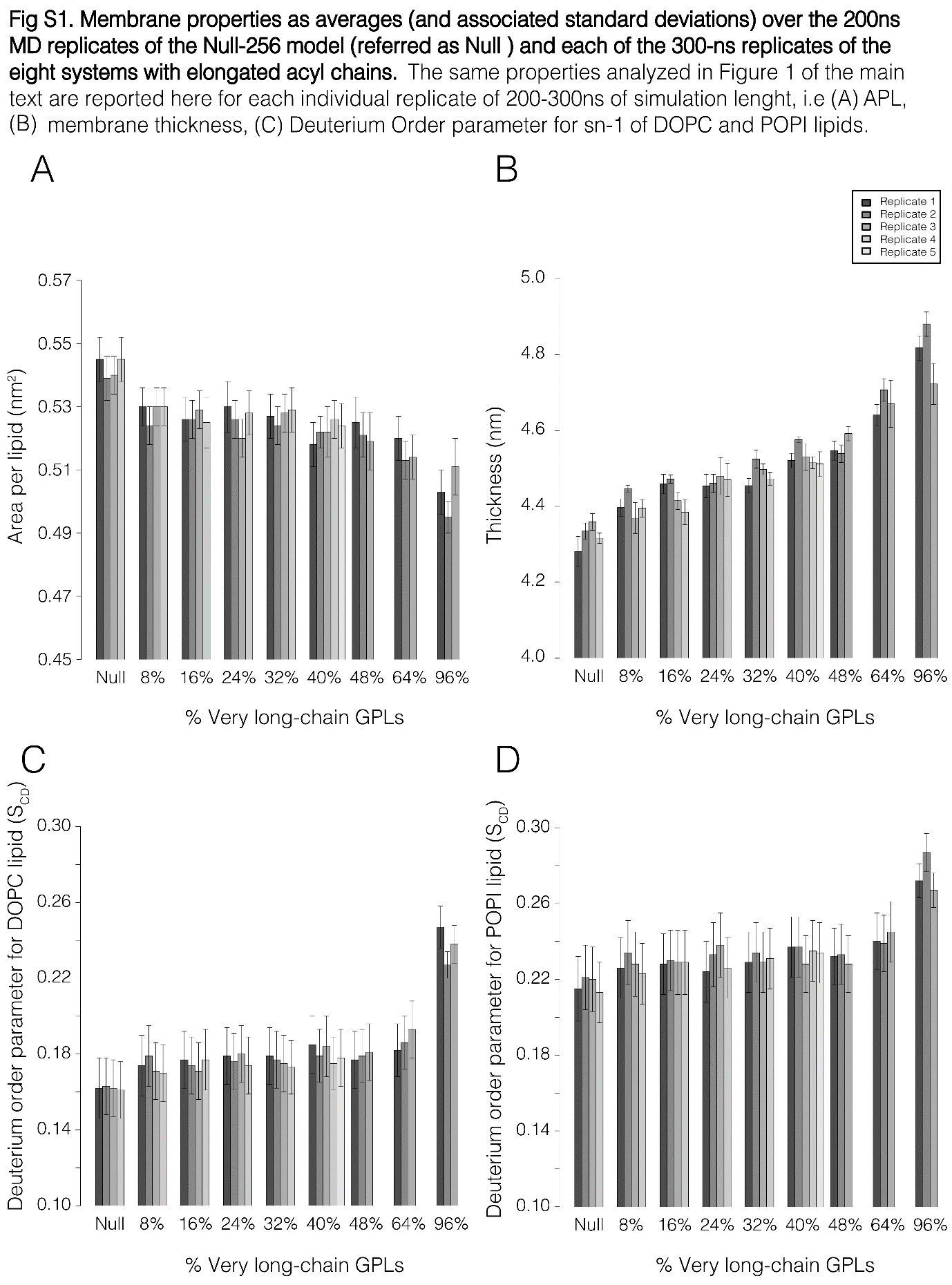


**S4 Membrane properties as averages (and associated standard deviations) for 300-ns or 500-ns simulations.**

The same properties analysed in figure 2 of the main text are reported here to compare the results using individual simulations of 300 or 500 ns of length, respectively (A, APL; B, membrane thickness; C, Deuterium Order parameter of DOPC lipids; and D, Deuterium order parameter of POPI lipids). For sake of clarity, we showed the example of replicate 2 for each system.


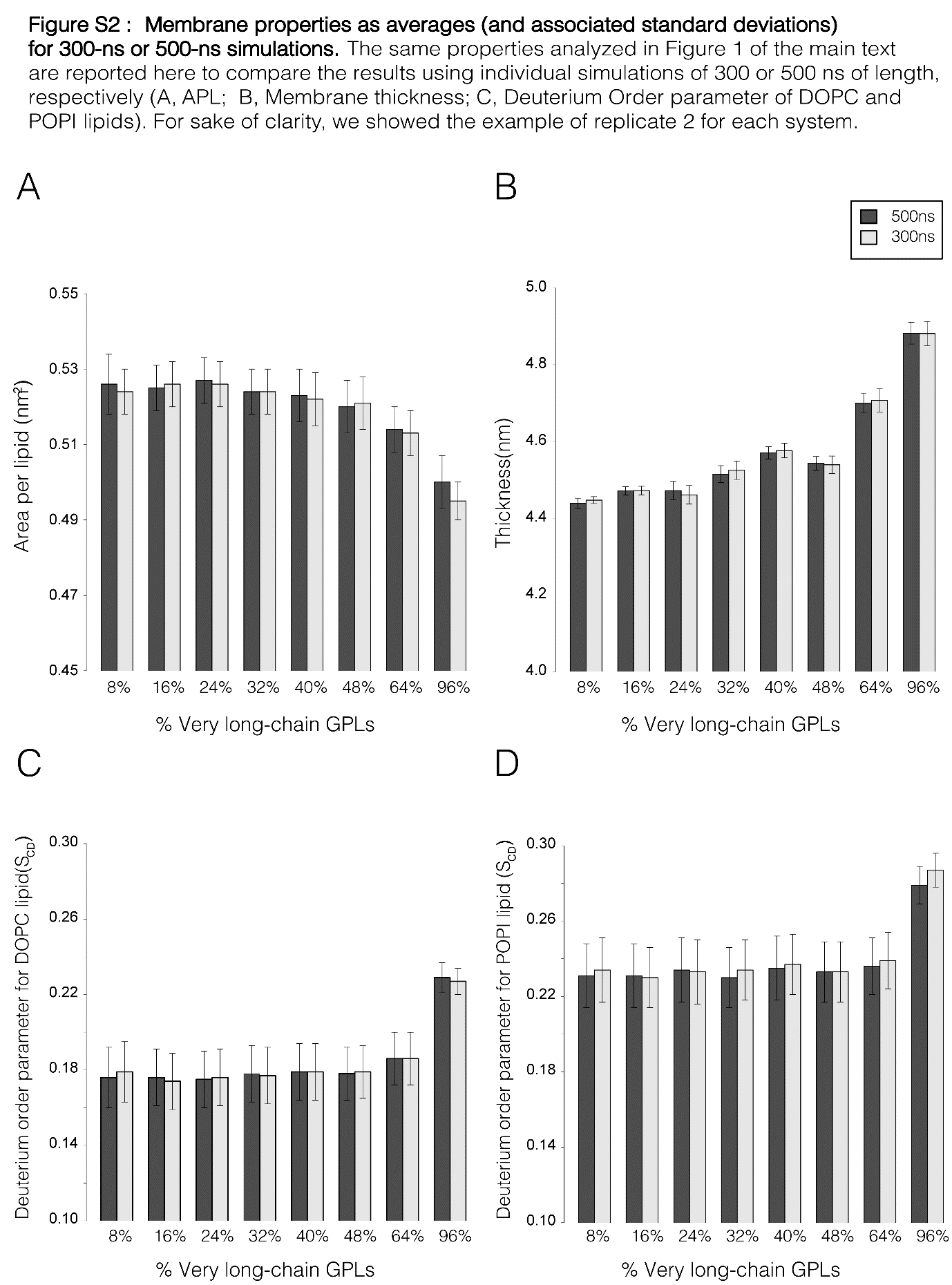


# S5 Boxplot of the structural parameters for the three replicates used in this study of each explored membrane systems

The same properties for the tree replicates used for figure 2, are represented here as a boxplot. The red square is the average value for the replicate, while the medians are shown as black lines. (A), Aera per lipid (APL); (B), membrane thickness (MT); (C) and (D) the deuterium parameter (S_CD_) of the *sn*1 acyl chains of the DOPC and POPI lipid respectively.


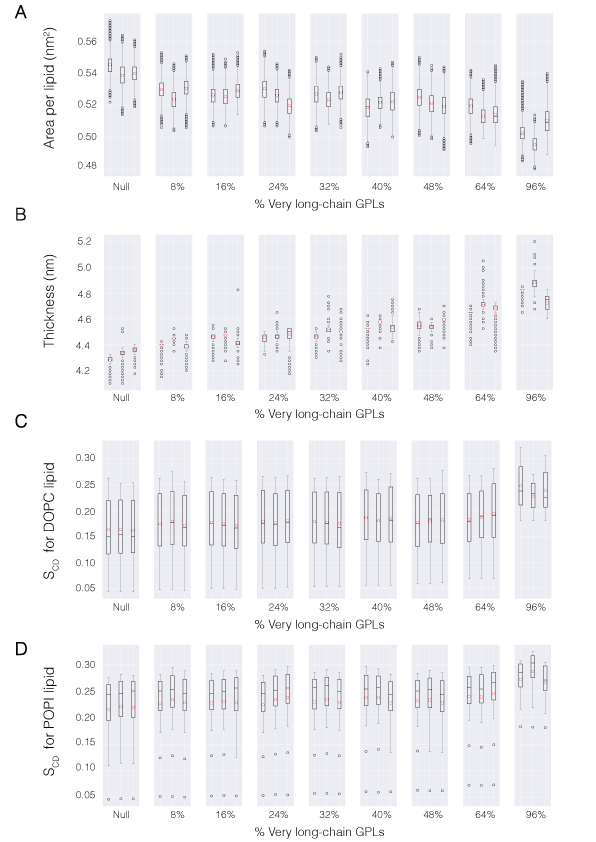


# S6 Codon optimised gene sequences

SEQ *Arabidopsis* *thaliana* FAE1: 1521 bp;

Composition 458 A; 281 C; 322 G; 460 T; 0 OTHER

Percentage: 30.1% A; 18.5% C; 21.2% G; 30.2% T; 0.0%OTHER

Molecular Weight (kDa): ssDNA: 470.34 dsDNA: 937.5

>*Arabidopsis thaliana* FAE1 sequence optimised for *Saccharomyces cerevisiae*

1 ATGACATCTG TAAATGTGAA GTTGTTGTAC AGATACGTTT TGACCAATTT CTTCAATTTA

61 TGTTTGTTCC CACTGACTGC GTTCTTAGCT GGTAAAGCCA GTAGATTAAC CATAAACGAT

121 TTGCACAATT TCTTATCCTA TCTTCAACAT AACCTGATAA CTGTGACTTT GCTATTTGCG

181 TTTACTGTGT TTGGTTTGGT ACTTTACATC GTAACCAGAC CAAATCCGGT ATACTTGGTT

241 GATTATAGCT GCTATCTACC TCCACCACAT CTGAAAGTCT CAGTGTCCAA AGTTATGGAT

301 ATCTTCTATC AGATTAGAAA GGCTGATACC TCATCAAGAA ATGTCGCATG TGATGATCCA

361 TCCTCTTTGG ACTTCTTGCG TAAAATTCAA GAAAGGTCTG GCCTAGGTGA TGAAACATAT

421 AGCCCAGAAG GGTTAATTCA TGTCCCTCCC AGGAAAACCT TTGCTGCTTC AAGAGAAGAG

481 ACAGAGAAGG TGATTATTGG GGCCTTAGAA AACCTGTTTG AAAACACGAA AGTTAATCCA

541 AGAGAAATTG GCATATTGGT TGTCAATTCC TCCATGTTTA ACCCTACACC TAGTCTTTCC

601 GCAATGGTGG TGAATACCTT CAAGCTTCGT AGTAATATCA AATCGTTTAA CCTTGGCGGT

661 ATGGGTTGTT CTGCTGGAGT TATCGCCATC GATTTGGCCA AAGACTTATT GCATGTACAT

721 AAGAACACAT ATGCATTAGT CGTTTCAACA GAGAATATTA CACAAGGTAT ATATGCAGGA

781 GAAAATCGTA GTATGATGGT GTCTAACTGT CTATTTAGGG TAGGAGGTGC TGCCATACTA

841 CTGAGCAACA AATCAGGGGA TAGGAGAAGA AGCAAATATA AGTTAGTTCA CACTGTCAGA

901 ACACATACTG GTGCTGATGA CAAATCCTTT AGATGTGTTC AACAGGAAGA TGACGAATCT

961 GGTAAGATAG GGGTTTGTTT ATCAAAAGAT ATTACGAACG TTGCAGGCAC TACCTTGACT

1021 AAGAATATAG CGACTTTGGG ACCCTTAATT TTACCGTTAA GCGAGAAGTT TCTATTCTTC

1081 GCTACTTTTG TAGCTAAGAA GTTGTTGAAA GATAAAATCA AACACTACTA TGTTCCTGAC

1141 TTTAAACTGG CCGTTGACCA CTTTTGCATT CATGCGGGTG GAAGAGCAGT TATAGATGAA

1201 TTAGAGAAGA ATCTTGGCCT ATCACCCATT GATGTCGAAG CTTCAAGAAG TACATTACAC

1261 AGATTTGGTA ACACGTCTAG TAGTTCGATT TGGTACGAGT TGGCATACAT TGAAGCAAAA

1321 GGCAGGATGA AGAAAGGTAA TAAGGCTTGG CAAATCGCAC TTGGTTCTGG TTTTAAATGC

1381 AATTCTGCTG TTTGGGTTGC ACTAAGAAAT GTCAAAGCTT CTGCCAACTC TCCTTGGCAA

1441 CATTGCATTG ACCGTTATCC AGTCAAGATC GATTCGGACT TGTCTAAGTC GAAAACGCAT

1501 GTACAGAATG GAAGATCATA A

SEQ *Arabidopsis thaliana* GPAT5: 1509 bp;

Composition 411 A; 297 C; 338 G; 463 T; 0 OTHER

Percentage: 27.2% A; 19.7% C; 22.4% G; 30.7% T; 0.0%OTHER

Molecular Weight (kDa): ssDNA: 466.43 dsDNA: 930.2

>*Arabidopsis thaliana* GPAT5 sequence optimised for *Saccharomyces cerevisiae*

1 ATGGTTATGG AACAAGCAGG CACTACATCA TATTCTGTAG TGAGTGAGTT CGAAGGTACG

61 ATCTTGAAGA ATGCCGACTC TTTTAGCTAT TTCATGCTTG TAGCGTTCGA AGCAGCTGGT

121 TTGATTAGAT TCGCCATTTT GCTATTTCTA TGGCCAGTAA TCACCTTACT AGACGTCTTT

181 AGTTACAAGA ATGCCGCCTT AAAACTGAAA ATCTTTGTCG CAACTGTTGG ATTGAGAGAA

241 CCTGAAATTG AAAGTGTAGC AAGAGCCGTT CTTCCAAAAT TTTATATGGA TGATGTTTCA

301 ATGGATACTT GGAGGGTCTT TTCAAGTTGT AAAAAACGTG TTGTAGTCAC AAGAATGCCA

361 CGTGTTATGG TTGAACGTTT CGCTAAAGAG CACTTGAGAG CTGATGAAGT AATAGGCACT

421 GAGTTAATTG TCAATAGGTT TGGTTTTGTT ACAGGATTAA TCAGAGAAAC CGATGTCGAT

481 CAATCAGCCT TGAACAGAGT GGCGAACTTA TTCGTAGGTA GAAGGCCACA ATTAGGCTTA

541 GGGAAGCCCG CCCTGACAGC ATCGACAAAC TTCTTGAGCT TATGCGAAGA GCACATTCAT

601 GCTCCAATTC CTGAGAATTA CAATCATGGT GATCAGCAAT TGCAGTTGAG GCCGTTACCC

661 GTGATATTTC ACGATGGAAG ATTGGTTAAG AGGCCGACGC CTGCAACTGC ACTTATTATA

721 CTGTTGTGGA TTCCTTTCGG TATAATACTA GCTGTTATAA GAATCTTTCT TGGGGCGGTT

781 CTGCCATTGT GGGCAACTCC ATACGTGTCT CAGATATTTG GAGGGCACAT AATTGTTAAA

841 GGCAAGCCAC CCCAACCACC AGCTGCCGGT AAATCGGGAG TCCTTTTCGT CTGCACGCAT

901 AGAACTCTTA TGGACCCCGT GGTTCTATCT TACGTTTTAG GTAGATCTAT TCCTGCCGTC

961 ACATATTCTA TCTCCAGATT GTCGGAGATT TTGTCCCCTA TACCAACGGT AAGATTGACT

1021 AGAATCAGGG ACGTAGACGC AGCTAAGATT AAGCAACAAT TGTCTAAAGG TGACCTAGTG

1081 GTGTGTCCAG AAGGTACAAC ATGTAGAGAA CCTTTTCTAC TAAGATTTTC TGCTCTTTTC

1141 GCAGAATTAA CTGATCGTAT TGTGCCGGTT GCGATGAACT ATAGAGTAGG CTTCTTTCAT

1201 GCAACTACAG CTAGAGGATG GAAAGGTTTG GACCCTATTT TTTTCTTTAT GAACCCTAGA

1261 CCGGTTTATG AGATTACCTT TCTGAATCAA TTGCCAATGG AAGCTACCTG TTCCAGCGGT

1321 AAATCCCCTC ATGATGTTGC TAATTACGTG CAACGTATCT TAGCTGCTAC CTTAGGTTTT

1381 GAATGCACCA ACTTCACAAG GAAAGATAAG TACAGAGTCT TAGCTGGTAA TGATGGCACT

1441 GTTTCCTATT TGTCACTGTT AGATCAGTTA AAGAAAGTTG TGTCAACCTT TGAACCCTGT

1501 TTGCATTAA

# S7 Plasmid maps


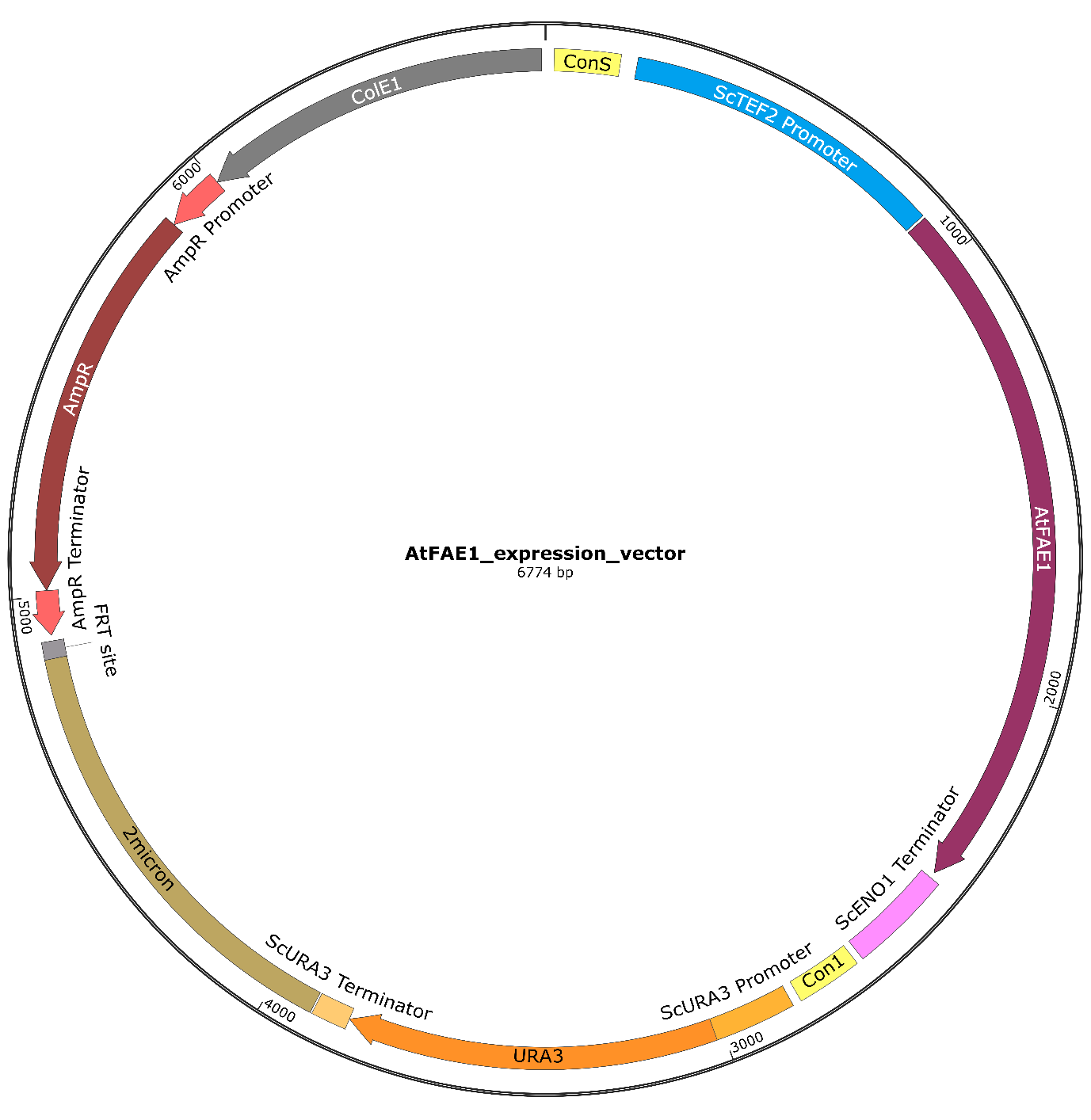


**Plasmid map of the *Arabidopsis thaliana* *FAE1* expression vector.** Plasmid map for the expression of the *A. thaliana* *FAE1* gene, which was codon optimised for expression in *Saccharomyces* *cerevisiae*. Plasmid remains in the yeast cell for expression of single gene (*FAE1*).


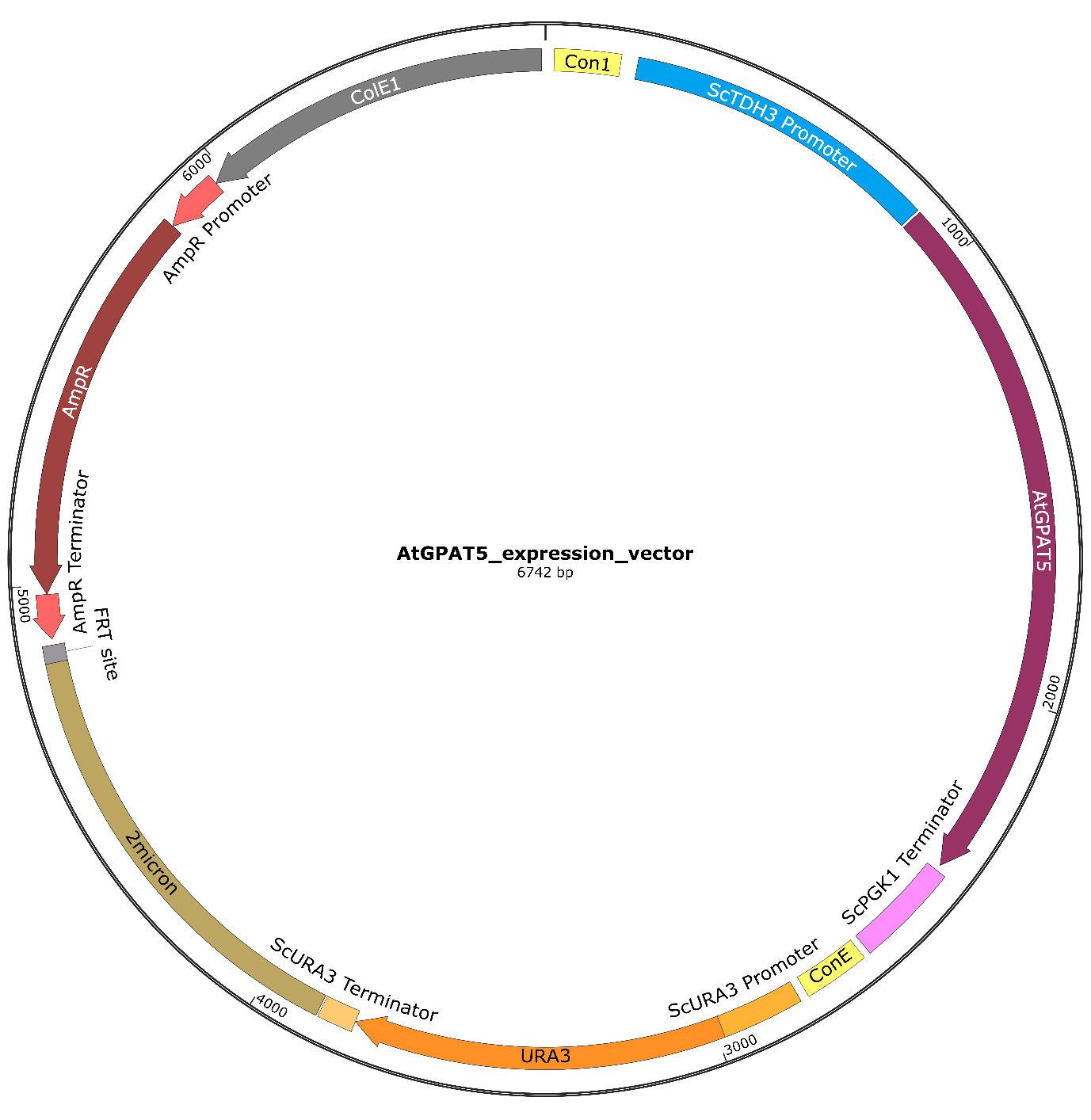


**Plasmid map of the *Arabidopsis thaliana* *GPAT5* expression vector.** Plasmid map for the expression of the *A. thaliana* *GPAT5* gene, which was codon optimised for expression in *Saccharomyces* *cerevisiae*. Plasmid remains in the yeast cell for expression of single gene (*GPAT5*).


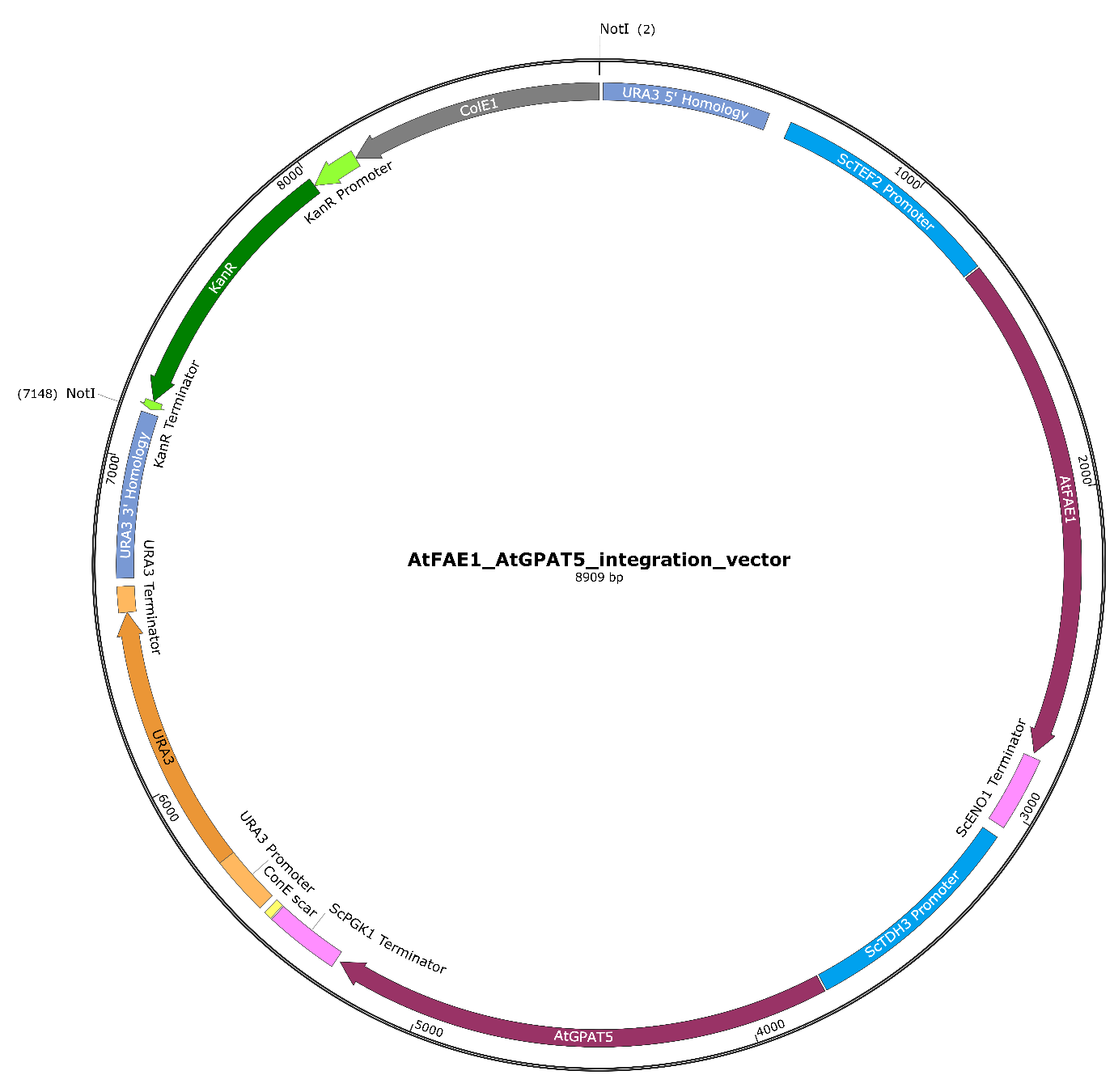


**Plasmid map of the *Arabidopsis thaliana* *FAE1* and *GPAT5* integration vector.** Plasmid map for the expression of the *A. thaliana* *FAE1* and *GPAT5* genes. The sequence of the genes was codon optimised for expression in *Saccharomyces* *cerevisiae*. Plasmid digested with NotI prior to the site-directed integration of the genes in the URA3 site in the *S. cerevisiae* genome for expression of the two genes.


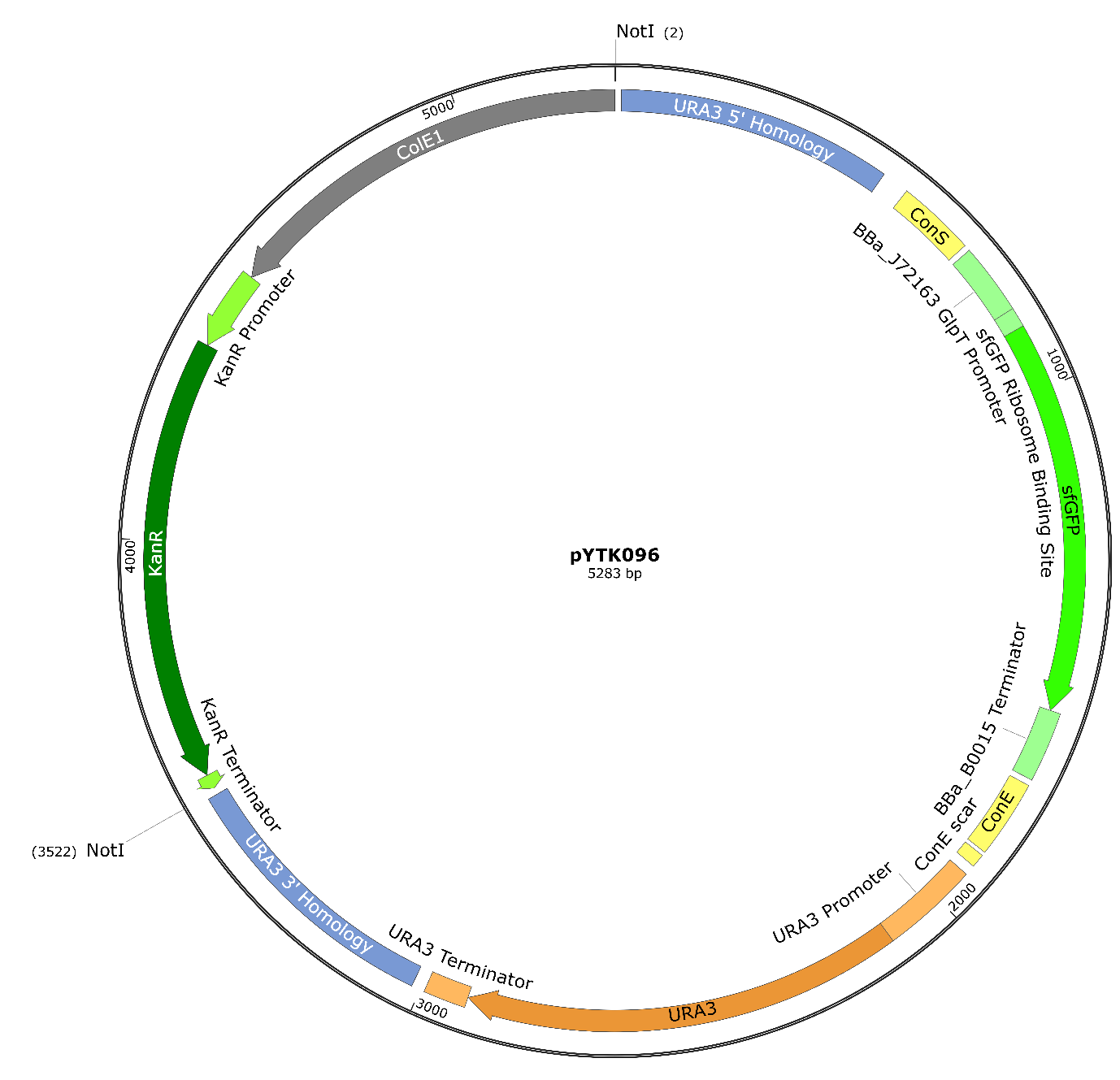


**Plasmid map of the vector used to create the control strain.** Plasmid digested with NotI prior to the site-directed integration into the URA3 site in the *S. cerevisiae* genome. The vector is part plasmid 96 of the MoClo kit (Addgene kit #1000000061).

# S8 Relative abundances lipids

**Relative abundance of fatty acid chains**

|  | | PM | CL | EE | TAG | Lyso-GPL |
| --- | --- | --- | --- | --- | --- | --- |
|  | FAE1 | 81.30% ± 1.312 | 1.12% ± 0.228 | 8.53% ± 0.216 | 8.20% ± 1.240 | 0.85% ± 0.101 |
|  | FAE1_GPAT5 | 70.95% ± 0.965 | 0.83% ± 0.118 | 5.24% ± 0.118 | 22.16% ± 0.791 | 0.82% ± 0.021 |
|  | GPAT5 | 74.45% ± 1.453 | 0.75% ± 0.135 | 7.72% ± 0.418 | 16.40% ± 1.234 | 0.69% ± 0.042 |
|  | Control | 67.96% ± 0.476 | 1.19% ± 0.104 | 7.53% ± 0.130 | 22.20% ± 0.507 | 1.11% ± 0.071 |

# S9 Single expression strains

**Glycerophospholipid acyl chain length of plasma membrane headgroups**. The figure depicts the combined chain length of the two acyl chains of glycerophospholipids obtained from total lipid analysis. The cumulative relative amounts of medium- (C24-31), long- (C32-36) and very-long- (C37-48) chain glycerophospholipids are shown in the insert. GPL, glycerophospholipid. The control strain depicted in blue, while the FAE_GPAT double expression strain is shown in orange. The single expression strains containing a free plasmid for expression of FAE and GPAT are shown in grey and yellow respectively.


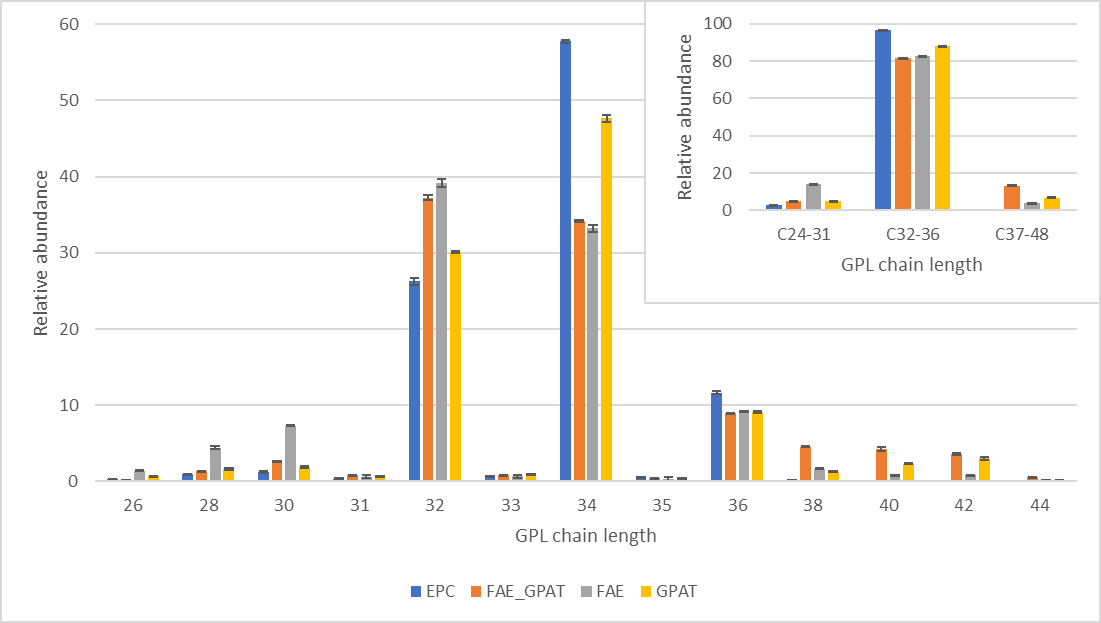


**Relative abundance of plasma membrane headgroups**. The figure shows the relative abundances of membrane-related lipid headgroups obtained from total lipid analysis, and the insert shows the cumulated amounts of different lipid classes. The controlstrain depicted in blue, while the FAE_GPAT double expression strain is shown in orange. The single expression strains containing a free plasmid for expression of FAE and GPAT are shown in grey and yellow respectively. CDP-DAG, cytidine diacylglycerol; Cer, Ceramides; DAG, diacylglycerol; GPL, glycerophospholipid; IPC, inositol phosphorylceramide; L-GPL, lyso-glycerophospholipid; M(IP)_2_C, mannosyl-di-(inositol phosphoryl) ceramide; MIPC, mannosyl-inositol phosphorylceramide; PA, phosphatidic acid; PC, phosphatidylcholine; PE, phosphatidylethanolamine; PI, phosphatidylinositol; PS, phosphatidylserine; SL, sphingolipid; TAG, triacylglycerol.


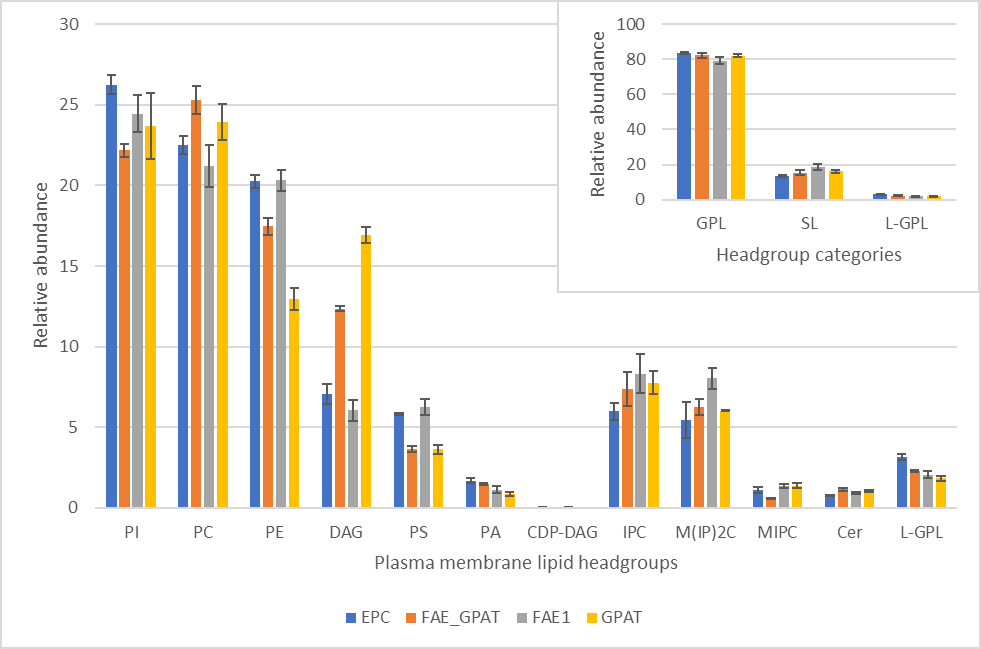


**Acetic acid uptake with an initial extracellular acetic acid concentration of 0.56 mM, pH 5.0.** Rational regression lines were calculated using MATLAB and the 90% confidence intervals are shown by the dotted lines. The average sample response is given at each time point measured. The control strain depicted in red, while the single expression strains containing a free plasmid for heterologous expression of FAE and GPAT are shown in blue and yellow respectively.


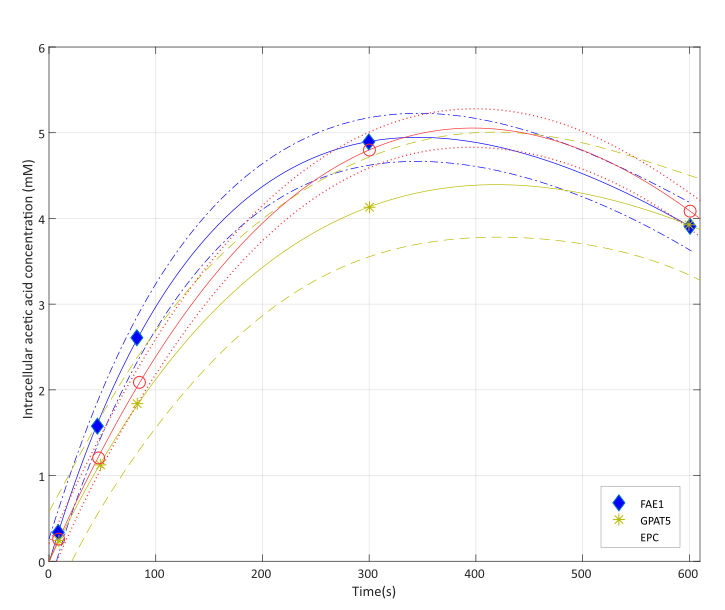


**Acetic acid uptake with an initial extracellular acetic acid concentration of 2.36 mM, pH 5.0.** Rational regression lines were calculated using MATLAB and the 90% confidence intervals are shown by the dotted lines. The average sample response is given at each time point measured. The control strain depicted in red, while the single expression strains containing a free plasmid for heterologous expression of FAE and GPAT are shown in blue and yellow respectively.


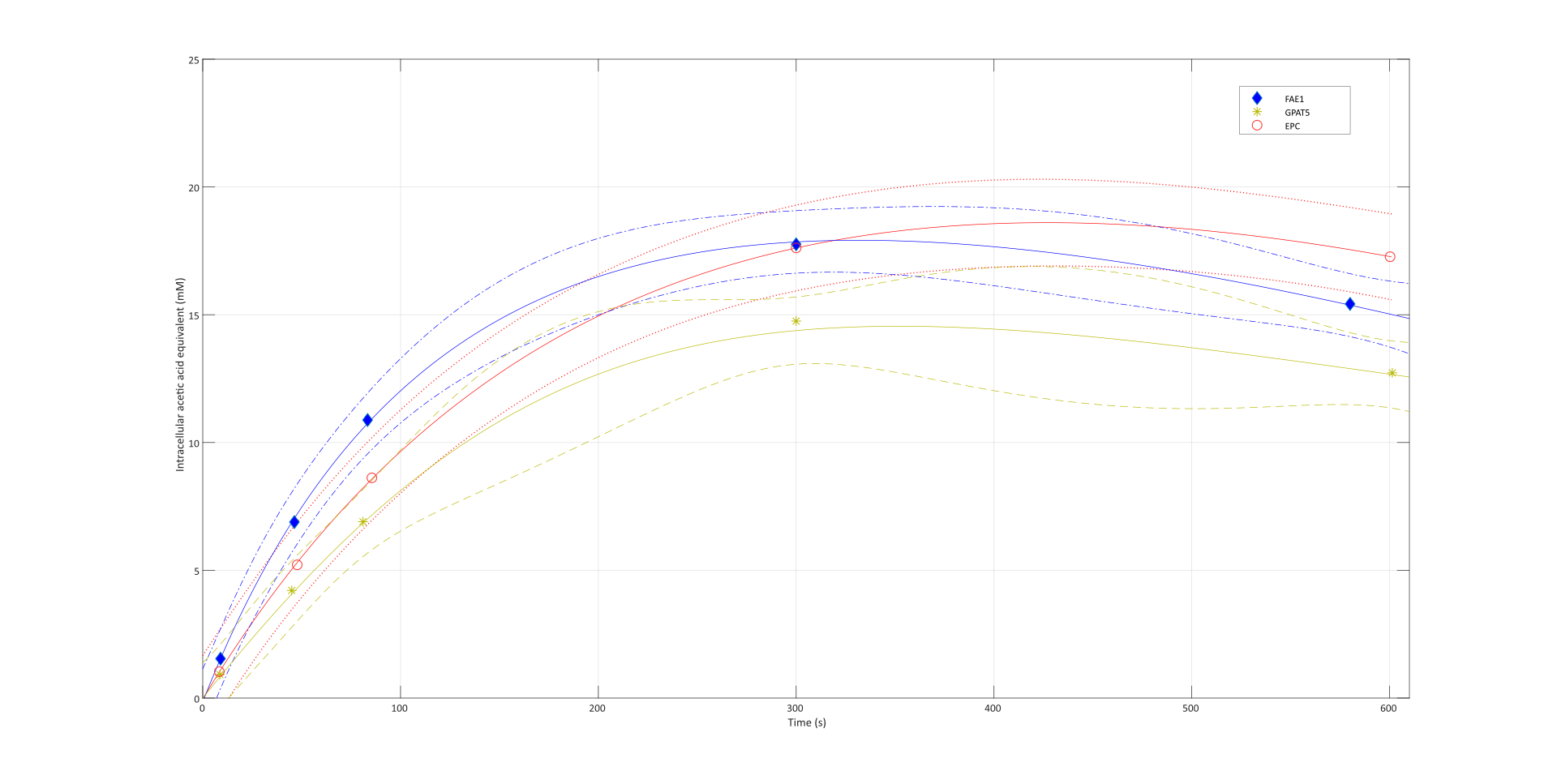


# S10 Growth profiler

**Growth profiler.** The control strain (orange) and the FAE1_GPAT5 double expression strain (blue) were grown in triplicate in the growth profiler at standard lab conditions. The experiment was performed in defined medium at pH5 with different concentrations of acetic acid. No significant difference can be seen between the growth rates of the two strains (see table below figures), the lag phase was inconsistent. Data on y-axis depicts calculated Log10 of the OD600 values (calculated from green values).

| Acetic acid concentration | Maximum specific growth rate ( Control strain) | Maximum specific growth rate ( FAE1_GPAT5) |
| --- | --- | --- |
| 0 g/L | 0.272 (σ 0.007) | 0.27 (σ 0.01) |
| 9 g/L | 0.14 (σ 0.01) | 0.15 (σ 0.02) |
| 13 g/L | 0.118 (σ 0.002) | 0.12 (σ 0.02) |

# S11 Screening lipid data

**Fatty acid methyl esterification (FAME) and Gas Chromatography (GC) analysis of EPC and FAE1_GPAT5.** The control strain (blue) and FAE1_GPAT5 double expression strain (green) total lipid chain lengths were analysed in triplicate using FAME and GC analysis. Much higher relative abundance of very-long-chain fatty acids is seen in the double expression transformant.


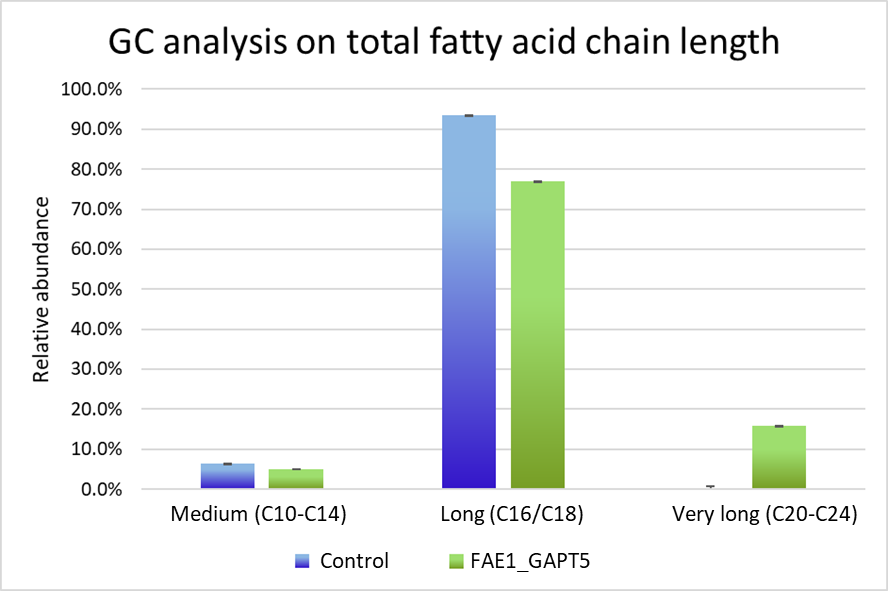


# S12 Additional data for uptake measurements

**Acetic acid uptake with an initial extracellular acetic acid concentration of 0.56 mM, pH 5.0.** Rational regression lines were calculated using MATLAB and the 90% confidence intervals are shown by the dotted lines. The average sample response is given at each time point measured. The highest intracellular concentration of acetic acid was observed in the empty plasmid control (EPC) strain (black), while the FAE1_GPAT5 strain showed lower maximum intracellular acetic acid concentrations (grey).


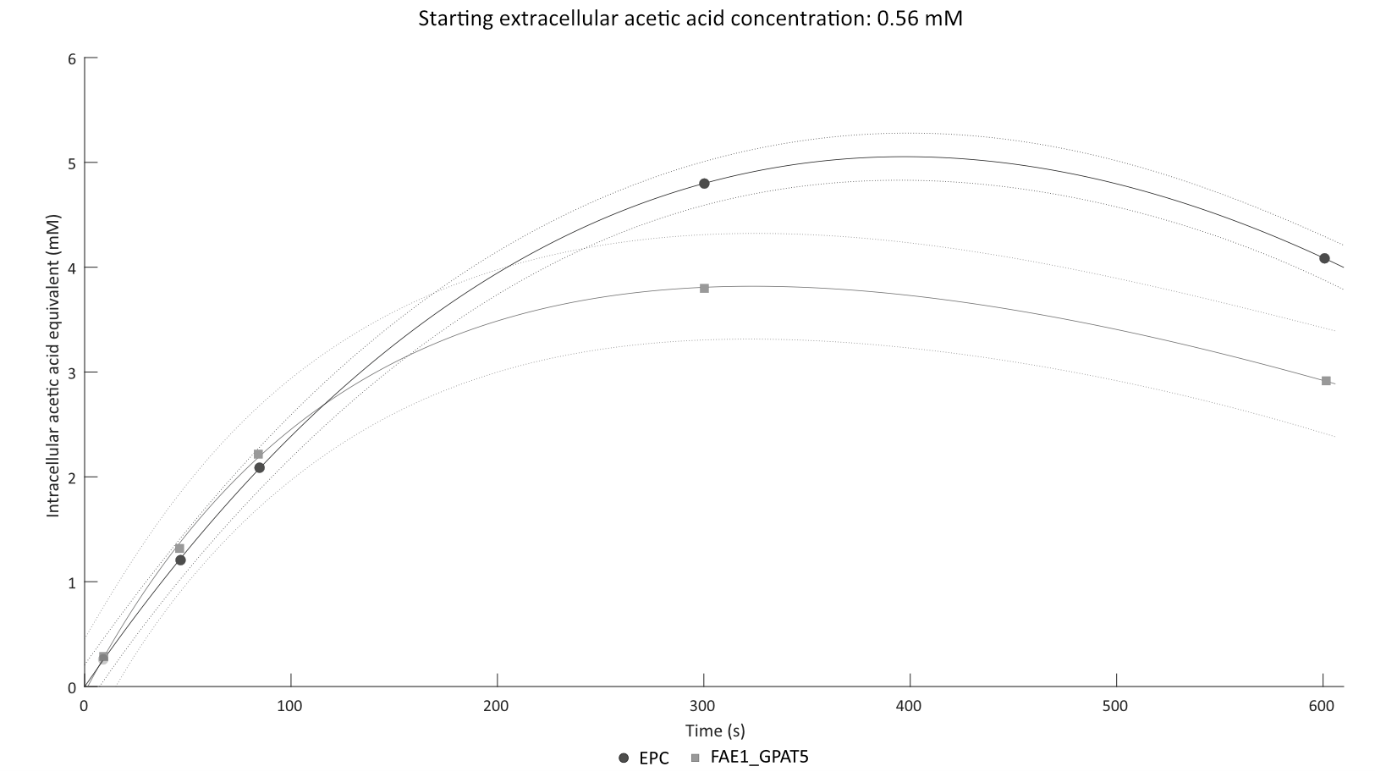


**Acetic acid uptake with an initial extracellular acetic acid concentration of 2.4 mM, pH 5.0.** Rational regression lines were calculated using MATLAB and the 90% confidence intervals are shown by the dotted lines. The average sample response is given at each time point measured. The highest intracellular concentration of acetic acid was observed in the empty plasmid control (EPC) strain (black), while the FAE1_GPAT5 strain showed lower maximum intracellular acetic acid concentrations (grey).


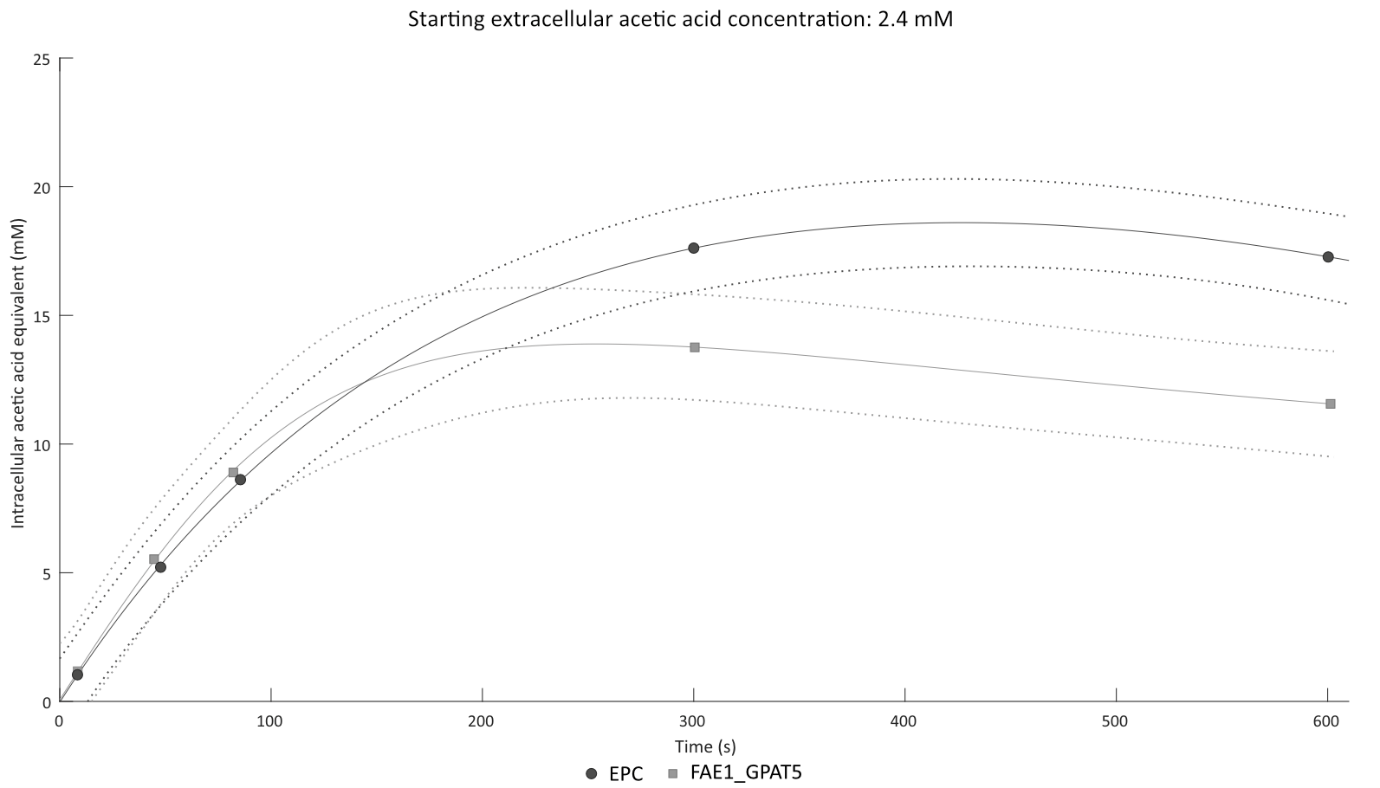


**Acetic acid uptake with an initial extracellular acetic acid concentration of 20 mM, pH 5.0.** Rational regression lines were calculated using MATLAB and the 90% confidence intervals are shown by the dotted lines. The average sample response is given at each time point measured. The highest intracellular concentration of acetic acid was observed in the empty plasmid control (EPC) strain (black), while the FAE1_GPAT5 strain showed lower maximum intracellular acetic acid concentrations (grey).


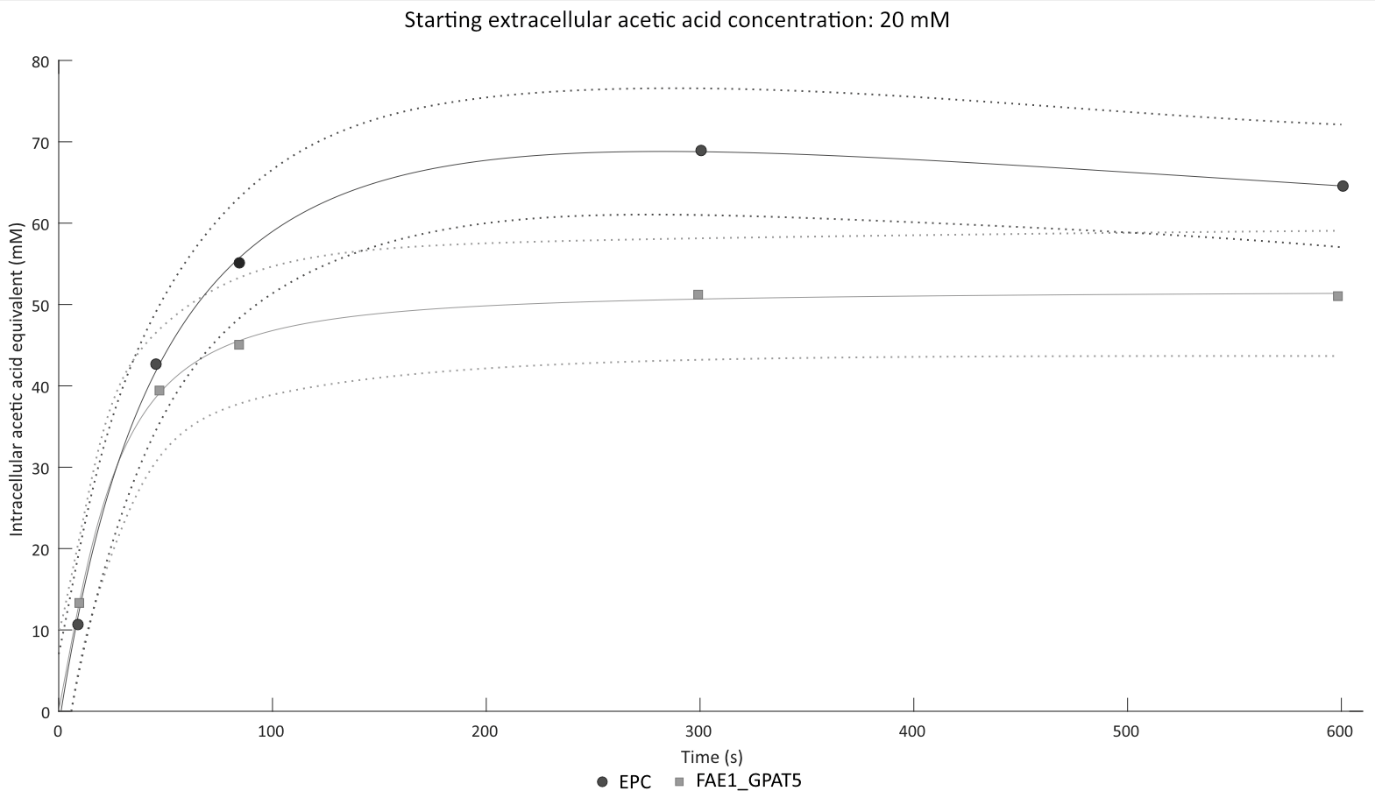

Supplement: Supplementary file 1 — Supplementary Information 1. [file 41598_2021_96757_MOESM1_ESM.docx]
